# KBG Syndrome: A Case Report and Longitudinal Assessment of Long‐Acting Recombinant Human Growth Hormone Therapy

**DOI:** 10.1002/ccr3.71744

**Published:** 2026-01-02

**Authors:** Hui Nan, Pu Zhang, Jing Qian

**Affiliations:** ^1^ Department of Pediatrics and Child Health Liaocheng People's Hospital Liaocheng China; ^2^ Department of Tumor Liaocheng People's Hospital Liaocheng China

**Keywords:** genetic disorders, growth retardation, KBG syndrome, long‐acting recombinant human growth hormone (rhGH) therapy, pediatric endocrinology

## Abstract

This case analysis examine**s** the clinical data, molecular genetic testing results, and 20‐month clinical data of long‐acting recombinant human growth hormone (rhGH) treatment in a child with KBG syndrome (KBGS). The child exhibited a c.1591delG frameshift mutation in the ANKRD11 gene associated with KBGS, a variant not previously reported, thereby enriching the genetic mutation spectrum of KBGS. Following treatment with long‐acting rhGH, the child showed significant improvement in height without adverse reactions.

## Summary

- This report details a KBG syndrome male child (with novel ANKRD11 c.1591delG mutation) who had short stature. 20‐month rhGH therapy effectively improved his height without adverse reactions, supporting rhGH‘s efficacy/safety for such cases.

## 1 Introduction

KBGS is a rare autosomal dominant genetic disorder first reported by Herrmann et al. [1] in 1975, named after the initials of the surnames of three initial affected families. The main characteristics of this syndrome include macrodontia of the upper central incisors, short stature, distinctive facial features, developmental delay or mild/moderate intellectual disability, delayed bone age, and epilepsy [2, 3, 4, 5, 6]. Growth hormone is applicable for children with short stature, growth hormone deficiency, Turner syndrome, etc. This article reviews and analyzes the clinical data of a child with KBGS who presented with short stature and discusses the efficacy and safety of long‐acting rhGH treatment.

## 2 Case History

This report presents a case of a 2‐year‐and‐9‐month‐old male child who was admitted to the *Department of Pediatrics and Child Health at Liaocheng People's Hospital* in Shandong Province on January 10, 2022, with the chief complaint of being shorter in stature compared to his peers. The study protocol was approved by the Medical Ethics Committee of Liaocheng People's Hospital in Shandong Province (Approval No.: 2024063), and informed consent was obtained from the patient's guardian.

The child was the firstborn, delivered via cesarean section at 38 weeks and 6 days of gestation, with no history of birth asphyxia or resuscitation. The birth weight was 3.3 kg, and the length was 45 cm. The mother, aged 26, had regular prenatal check‐ups. Ultrasound examination at 12–15 weeks of gestation showed increased nuchal translucency (NT) thickness. Noninvasive DNA testing results were normal. Subsequent chorionic villus sampling for chromosomal microarray analysis (CMA), amniocentesis for karyotype analysis, and testing for chromosomal aneuploidies and 100 Kb copy number variations (CNVs) revealed no abnormalities. A color Doppler ultrasound at 23 weeks of gestation showed increased orbital distance and absence of the nasal bone. Gross motor development was comparable to peers: the child could roll over at 4 months, sit steadily at 7 months, stand at 1 year, and walk at 1 year and 1 month. Language development was delayed, with the child consciously calling parents at 2 years and speaking phrases at 2 years and 9 months. The child had a history of otitis media twice, with normal hearing. The patient had no history of epilepsy or febrile seizures. Both parents were healthy; the mother was 158 cm tall, and the father was 170 cm tall. They were not consanguineously married, and there was no family history of inherited metabolic diseases. Physical examination: height 83.0 cm (< P3), weight 10.3 kg (< P3), clear consciousness, responsive, and stable breathing. The child had a short stature, with no jaundice or rash on the skin and mucous membranes. The forehead was prominent, with wide‐set eyes, ptosis, upward slanting of the outer canthus, a low nasal bridge, upturned nasal tip, and a high‐arched palate. The fingers were short and thick, with the fifth finger having a short middle phalanx and radial deviation, and a simian crease on the right hand. There was no chest deformity, rib flaring, cryptorchidism, or hydrocele. The heart, lungs, and abdomen were normal, and the limbs were freely movable.

The results of the neuropsychological development assessment in children showed: a mental age of 28 months, a developmental quotient of 83; gross motor skills equivalent to 24 months; fine motor skills equivalent to 33 months; language skills equivalent to 21 months; personal‐social skills equivalent to 31.5 months. Routine blood and urine tests were normal; liver and kidney functions were generally normal; thyroid function was normal; thyroid antibody tests were normal; adrenocorticotropic hormone (ACTH) was normal; lactate levels were normal; insulin‐like growth factor‐1 (IGF‐1) was 118 ng/mL (reference range: 51–303 ng/mL); the peak of the growth hormone stimulation test was 5.75 ng/mL (30 min); cranial MRI showed no abnormal signals in the brain parenchyma. Combined with the child's height below the 3rd percentile (P3), bone age lagging by 2.25 years, peak growth hormone stimulation test below 10 ng/mL, and low IGF‐1 levels, a definitive diagnosis of growth hormone deficiency (GHD) was made.

**FIGURE 1:**
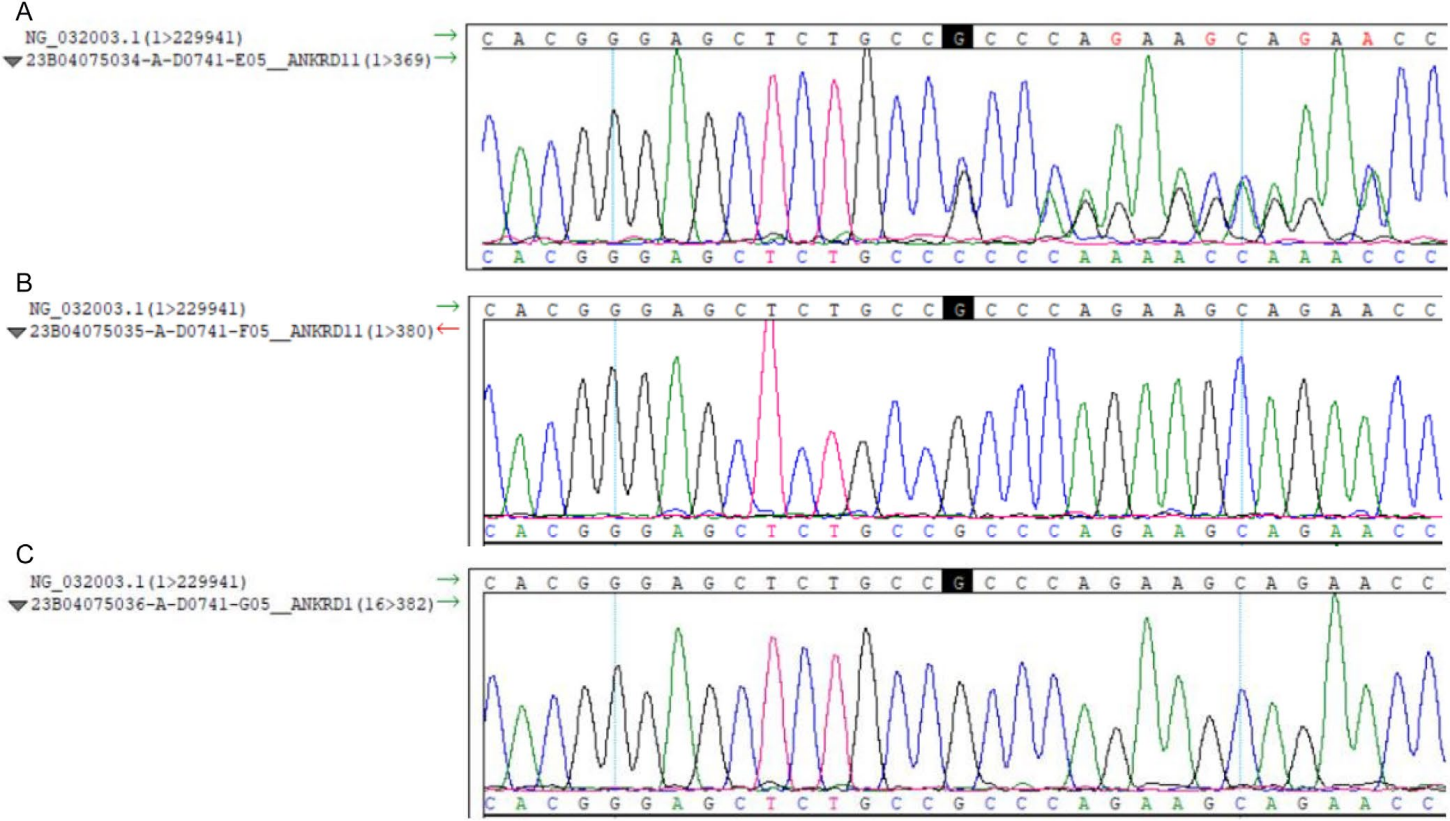
ANKRD11 exon 10 sequencing results: (A) proband with c.1591delG frameshift mutation; (B) father with wild‐type sequence; (C) mother with wild‐type sequence (red arrow = mutation site).

After obtaining informed consent from the child's guardian, peripheral blood samples were collected from the child and his parents for whole‐genome high‐throughput sequencing. The results revealed a c.1591delG frameshift mutation in exon 10 of the ANKRD11 gene associated with KBGS, leading to premature termination of protein coding (p.Ala531Profs*43). According to the ACMG guidelines, this variant was classified as a pathogenic variant, PVS1 + PS2_Moderate+PM2. This variant has not been previously reported. According to the ACMG guidelines (Appendix S1), the variant was classified as pathogenic. Familial verification indicated that this variant is a de novo mutation, as neither parent carried the variant. The child was ultimately diagnosed with ANKRD11 gene‐related KBGS, which is an autosomal dominant inheritance disorder (Figure 1).

**FIGURE 2:**
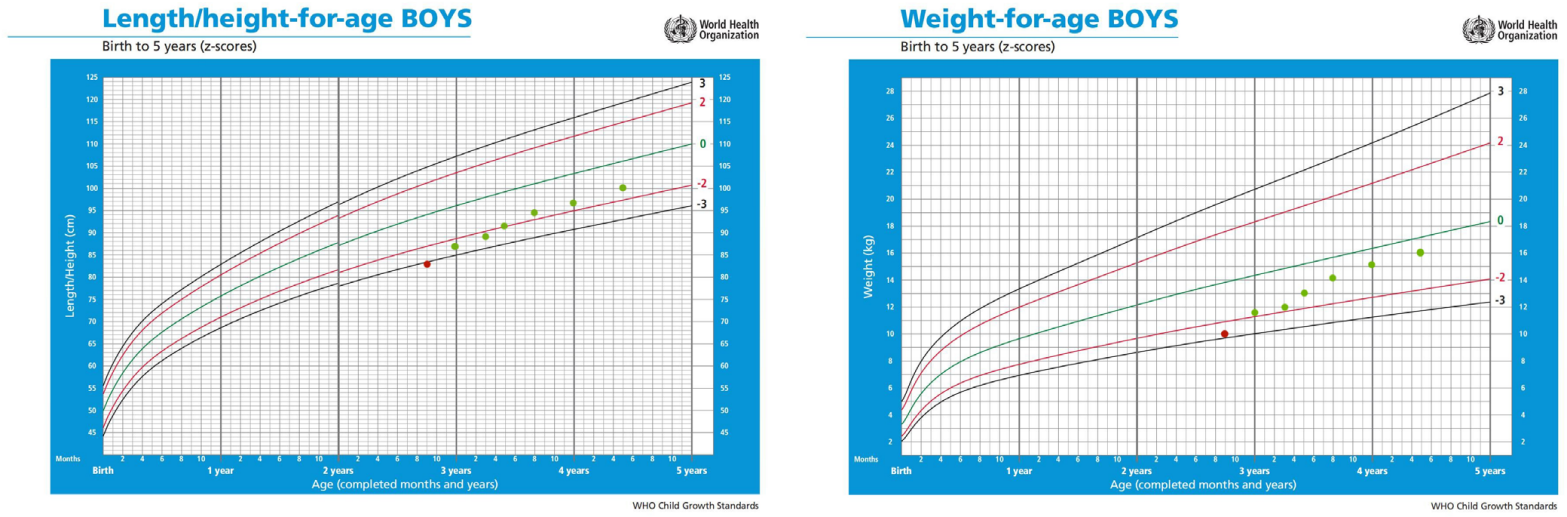
Shifts in length/height and weight SDS in this child following long‐acting growth hormone administration (red: pre; green: post).

After diagnosis, long‐acting rhGH therapy was immediately initiated at a dose of 0.18 mg/(kg·w) subcutaneously, starting at the age of 2 years and 9 months, with a height of 83 cm and a height standard deviation score (HtSDS) of −3.18. During treatment, IGF‐1 levels fluctuated between 118 and 302 ng/mL; monitoring of blood routine, liver and kidney functions, thyroid function, fasting blood glucose, and insulin showed no abnormalities. After 20 months of long‐acting rhGH treatment, height increased by 17.5 cm, with an average growth rate of 10.5 cm/year, reaching a height of 100.5 cm at 4 years and 5 months, with an HtSDS of −1.61 (Table 1). From the growth curve, it was evident that the child's height and weight had shown significant improvement (Figure 2).

**TABLE 1:** Physical monitoring, IGF‐1 levels, long‐acting rhGH dosage, and height standard deviation score (Ht SDS) of pediatric patients.

Date	Age	Height (cm)	Weight (kg)	IGF‐1 levels (ng/mL)	Long‐acting rhGH dosage (mg)	Ht SDS
2022‐01‐20	2 years and 9 months	83	10.3	118.00	1.8	−3.18
2022‐04‐20	2 years	86.8	11.7	213.12	2.0	−2.63
2022‐07‐21	3 years and 3 months	88.4	12.0	223.12	2.0	−2.71
2022‐10‐03	3 years and 5 months	91.2	13	204.00	2.5	−2.26
2023‐01‐15	3 years and 8 months	94.5	14.3	302.17	2.5	−1.87
2023‐05‐07	4 years	97.5	15.2	230.7	2.5	−1.69
2023‐10‐07	4 years and 5 months	100.5	16	280.2	2.5	−1.61

## 3 Discussion

KBGS is caused by mutations in the ANKRD11 gene or deletions in the 16q24.3 region [7]. The incidence of this disease has not been clearly defined, with over 200 cases reported globally, and the male‐to‐female ratio is approximately 1.5:1. Due to mild symptoms or incomplete recognition in some patients, the actual incidence may be underestimated, with female patients generally exhibiting milder symptoms [4, 8, 9]. Among pathogenic mutations, single nucleotide mutations and small insertions/deletions within the ANKRD11 gene account for approximately 71%, while deletions of 16q24.3 containing ANKRD11 account for about 29%, with no mutation hotspots identified [5]. In patients with ANKRD11 gene mutations, 66% are de novo mutations, and in patients with 16q24.3 deletions, 75% are de novo mutations, with the remaining cases being autosomal dominant inheritance [4]. This case involves a male child, with genetic testing revealing a de novo variant caused by a single nucleotide deletion in the ANKRD11 gene, a variant at this site not previously reported.

The genetic mutation spectrum and phenotypic spectrum of KBGS exhibit complex diversity. The clinical diagnostic criteria proposed in 2016 [2] include the following major indicators: macrodontia of the upper central incisors, short stature, recurrent otitis media with or without hearing loss, and first‐degree relatives with KBGS; minor indicators include short fingers or related hand abnormalities, epilepsy, cryptorchidism, feeding difficulties, abnormal palatal arch, autism, large anterior fontanelle or delayed closure. For patients presenting with developmental delay, learning difficulties, or significant behavioral abnormalities, a clinical diagnosis of KBGS can be made if they exhibit two major indicators or one major indicator and two minor indicators.

The patient in this case presented with short stature and distinctive facial features, including a prominent forehead, hypertelorism, ptosis, upward slanting palpebral fissures, a low nasal bridge, an upturned nasal tip, and a high‐arched palate. The hand characteristics included short and thick fingers, with the fifth finger showing a short middle phalanx and radial clinodactyly, and a single palmar crease on the right hand. The patient had a history of two episodes of otitis media, but hearing was not impaired, which is consistent with the clinical diagnostic criteria for KBGS.

Most KBGS patients exhibit some degree of developmental delay, particularly in language development [9]. In this case, the patient's language development was delayed by 12 months compared to the actual age. Approximately half of the patients experience seizures or abnormal EEG findings without seizures [2], with the onset age ranging from infancy to adolescence. Antiepileptic drugs can effectively control the symptoms, and most patients experience symptom relief after adolescence. The patient in this case has not yet experienced seizures, and further EEG examination is required to rule out abnormal EEG findings. Maria‐loanna Stefanou reported a patient with KBGS, which has brain magnetic resonance imaging (MRI) demonstrated multiple gray‐matter (GM) heterotopias and hypoplasia of the corpus callosum [10]. In this case, the boy's cranial MRI showed no abnormal signals in the brain parenchyma. It can be seen that ANKRD11 mutations may be related to brain development, and in addition to electroencephalography, cranial magnetic resonance imaging is also a necessary auxiliary examination.

In previously reported cases of KBGS, ANKRD11 gene mutations were predominantly located in exon 9 [8, 11]. In this case, the gene mutation was located in exon 10, further enriching the spectrum of gene mutations associated with KBGS.

The ANKRD11 gene is located on the long arm of chromosome 16 and encodes the ankyrin repeat domain‐containing protein 11. This protein comprises four functional domains: the ankyrin repeat domain, a transcriptional activation domain, and two transcriptional repression domains. ANKRD11 is primarily localized in the nucleus, where it directly interacts with the tumor suppressor protein TP53, participating in cell cycle regulation [12, 13]. Additionally, as a co‐regulatory factor, ANKRD11 inhibits ligand‐dependent transcriptional activation by interacting with the p160 coactivator and various HDAC corepressors [14, 15]. Studies have demonstrated that ANKRD11 can influence neurodevelopmental processes by regulating histone acetylation and gene expression [16].

Currently, there are no formal clinical management guidelines for KBGS. For the short stature of this child, evidence has shown that long‐acting rhGH treatment is safe and effective [17]. During long‐acting rhGH treatment, the child's average annual growth rate reached 10.5 cm, indicating effective treatment and good height benefits. Monitoring indicators such as blood routine, liver and kidney function, IGF‐1, thyroid function, and fasting blood glucose and insulin were all within the normal range, and the evaluation showed that long‐acting rhGH treatment was safe. For the child's developmental delay, especially the lag in language ability, cognitive rehabilitation training was also conducted, and significant progress was made in all developmental areas. Currently, the child has entered kindergarten and is able to engage in normal learning and life.

A 20‐month follow‐up revealed significant height growth in the pediatric patient without any adverse reactions. According to the parents, substantial progress has been achieved through our regular and standardized treatment regimen, which is highly encouraging.

In summary, KBGS is a rare genetic disease characterized by short stature, special facial features, macrodontia, and developmental delay. Long‐acting rhGH treatment can effectively improve the adult height of the child and is safe; cognitive rehabilitation training helps alleviate developmental delay and enhance cognitive ability. This study reports a new variant of the ANKRD11 gene, which not only enriches the gene variant database of KBGS but also provides new clinical evidence and rehabilitation strategies for the treatment of the disease and the improvement of the quality of life of the child.

## Author Contributions

**Hui Nan:** data curation, formal analysis, investigation, methodology, project administration, resources, writing – original draft, writing – review and editing. **Pu Zhang:** investigation, project administration. **Jing Qian:** investigation, resources.

## Funding

The authors have nothing to report.

## Consent

Written informed consent was obtained from the patient to publish this report in accordance with the journal's patient consent policy.

## Conflicts of Interest

The authors declare no conflicts of interest.

## Supporting information


**Appendix S1:** ccr371744‐sup‐0001‐AppendixS1.pdf.

## Data Availability

The authors have nothing to report.

## References

[1] J. Herrmann , P. D. Pallister , W. Tiddy , et al., “The KBG Syndrome—A Syndrome of Short Stature, Characteristic Facies, Mental Retardation, Macrodontia and Skeletal Anomalies,” Birth Defects Original Article Series 11 (1975): 7–18. 1218237

[2] K. Low , T. Ashraf , N. Canham , et al., “Clinical and Genetic Aspects of KBG Syndrome,” American Journal of Medical Genetics, Part A 170 (2016): 2835–2846, 10.1002/ajmg.a.37842. 27667800 PMC5435101

[3] A. Goldenberg , F. Riccardi , A. Tessier , et al., “Clinical and Molecular Findings in 39 Patients With KBG Syndrome Caused by Deletion or Mutation of ANKRD11,” American Journal of Medical Genetics, Part A 170 (2016): 2847–2859, 10.1002/ajmg.a.37878. 27605097

[4] D. Morel Swols , J. Foster , and M. Tekin , “KBG Syndrome,” Orphanet Journal of Rare Diseases 12 (2017): 183, 10.1186/s13023-017-0736-8. 29258554 PMC5735576

[5] E. Scarano , M. Tassone , C. Graziano , et al., “Novel Mutations and Unreported Clinical Features in KBG Syndrome,” Molecular Syndromology 10 (2019): 130–138, 10.1159/000496172. 31191201 PMC6528090

[6] L. C. M. van Dongen , E. Wingbermühle , W. Oomens , et al., “Intellectual Profiles in KBG‐Syndrome: A Wechsler Based Case‐Control Study,” Frontiers in Behavioral Neuroscience 11 (2017): 248, 10.3389/fnbeh.2017.00248. 29311865 PMC5742227

[7] D. Wang , P. Lai , and X. Li , “Analysis of ANKRD11 Gene Variant in a Family Affected With KBG Syndrome,” Zhonghua Yi Xue Yi Chuan Xue Za Zhi 37 (2020): 1029–1031, 10.3760/cma.j.cn511374-20190907-00462. 32820523

[8] A. Sirmaci , M. Spiliopoulos , F. Brancati , et al., “Mutations in ANKRD11 Cause KBG Syndrome, Characterized by Intellectual Disability, Skeletal Malformations, and Macrodontia,” American Journal of Human Genetics 89 (2011): 289–294, 10.1016/j.ajhg.2011.06.007. 21782149 PMC3155157

[9] A. Lo‐Castro , F. Brancati , M. C. Digilio , et al., “Neurobehavioral Phenotype Observed in KBG Syndrome Caused by ANKRD11 Mutations,” American Journal of Medical Genetics, Part B: Neuropsychiatric Genetics 162B (2013): 17–23, 10.1002/ajmg.b.32113. 23184435

[10] M. I. Stefanou , V. K. Katsaros , G. Pepe , et al., “Early‐Onset Parkinson's Disease in a Patient With a De Novo Frameshift Variant of the ANKRD11 Gene and KBG Syndrome,” Journal of Clinical Neurology 21, no. 2 (2025): 153–155, 10.3988/jcn.2024.0454. 40065458 PMC11896747

[11] S. Sacharow , D. Li , Y. S. Fan , and M. Tekin , “Familial 16q24.3 Microdeletion Involving ANKRD11 Causes a KBG‐Like Syndrome,” American Journal of Medical Genetics. Part A 158A (2012): 547–552, 10.1002/ajmg.a.34436. 22307766

[12] K. Walz , D. Cohen , P. M. Neilsen , et al., “Characterization of ANKRD11 Mutations in Humans and Mice Related to KBG Syndrome,” Human Genetics 134 (2015): 181–190, 10.1007/s00439-014-1509-2. 25413698

[13] P. M. Neilsen , K. M. Cheney , C. W. Li , et al., “Identification of ANKRD11 as a p53 Coactivator,” Journal of Cell Science 121 (2008): 3541–3552, 10.1242/jcs.026351. 18840648

[14] A. Zhang , C. W. Li , and J. D. Chen , “Characterization of Transcriptional Regulatory Domains of Ankyrin Repeat Cofactor‐1,” Biochemical and Biophysical Research Communications 358 (2007): 1034–1040, 10.1016/j.bbrc.2007.05.017. 17521611 PMC1950474

[15] A. Zhang , P. L. Yeung , C. W. Li , et al., “Identification of a Novel Family of Ankyrin Repeats Containing Cofactors for p160 Nuclear Receptor Coactivators,” Journal of Biological Chemistry 279 (2004): 33799–33805, 10.1074/jbc.M403997200. 15184363

[16] D. Gallagher , A. Voronova , M. A. Zander , et al., “Ankrd11 Is a Chromatin Regulator Involved in Autism That Is Essential for Neural Development,” Developmental Cell 32 (2015): 31–42, 10.1016/j.devcel.2014.11.031. 25556659

[17] N. Reynaert , C. W. Ockeloen , L. Sävendahl , et al., “Short Stature in KBG Syndrome: First Responses to Growth Hormone Treatment,” Hormone Research in Pædiatrics 83 (2015): 361–364, 10.1159/000380908. 25833229

