# Supplementary material for: KBG Syndrome: A Case Report and Longitudinal Assessment of Long‐Acting Recombinant Human Growth Hormone Therapy
Source: Clin Case Rep. 2026 Jan 2;14(1):e71744. doi: 10.1002/ccr3.71744 (PMC12759011; doi:10.1002/ccr3.71744)
Supplement: Supplementary file 1 — Appendix S1: ccr371744‐sup‐0001‐AppendixS1.pdf. [file CCR3-14-e71744-s001.pdf]

Standards and guidelines for the interpretation of sequence variants: a joint consensus recommendation of the American College of Medical Genetics and Genomics and the Association for Molecular Pathology

Sue Richards, PhD[1], Nazneen Aziz, PhD[2,16], Sherri Bale, PhD[3], David Bick, MD[4], Soma Das, PhD[5], Julie Gastier-Foster, PhD[6,7,8], Wayne W. Grody, MD, PhD[9,10,11], Madhuri Hegde, PhD[12], Elaine Lyon, PhD[13], Elaine Spector, PhD[14], Karl Voelkerding, MD[13] and Heidi L. Rehm, PhD[15]; on behalf of the ACMG Laboratory Quality Assurance Committee

Disclaimer: These ACMG Standards and Guidelines were developed primarily as an educational resource for clinical laboratory geneticists to help them provide quality clinical laboratory services. Adherence to these standards and guidelines is voluntary and does not necessarily assure a successful medical outcome. These Standards and Guidelines should not be considered inclusive of all proper procedures and tests or exclusive of other procedures and tests that are reasonably directed to obtaining the same results. In determining the propriety of any specific procedure or test, the clinical laboratory geneticist should apply his or her own professional judgment to the specific circumstances presented by the individual patient or specimen. Clinical laboratory geneticists are encouraged to document in the patient's record the rationale for the use of a particular procedure or test, whether or not it is in conformance with these Standards and Guidelines. They also are advised to take notice of the date any particular guideline was adopted and to consider other relevant medical and scientific information that becomes available after that date. It also would be prudent to consider whether intellectual property interests may restrict the performance of certain tests and other procedures.

The American College of Medical Genetics and Genomics (ACMG) previously developed guidance for the interpretation of sequence variants.[1] In the past decade, sequencing technology has evolved rapidly with the advent of high-throughput next-generation sequencing. By adopting and leveraging next-generation sequencing, clinical laboratories are now performing an ever-increasing catalogue of genetic testing spanning genotyping, single genes, gene panels, exomes, genomes, transcriptomes, and epigenetic assays for genetic disorders. By virtue of increased complexity, this shift in genetic testing has been accompanied by new challenges in sequence interpretation. In this context the ACMG convened a workgroup in 2013 comprising representatives from the ACMG, the Association for Molecular Pathology (AMP), and the College of American Pathologists to revisit and revise the standards and guidelines for the interpretation of sequence variants. The group consisted of clinical laboratory directors and clinicians. This report represents expert opinion of the workgroup with input from ACMG, AMP, and College of American Pathologists stakeholders. These recommendations primarily apply to the breadth of genetic tests used in clinical laboratories, including genotyping, single genes, panels, exomes, and genomes. This report recommends the use of specific standard terminology—"pathogenic," "likely pathogenic," "uncertain significance," "likely benign," and "benign"—to describe variants identified in genes that cause Mendelian disorders. Moreover, this recommendation describes a process for classifying variants into these five categories based on criteria using typical types of variant evidence (e.g., population data, computational data, functional data, segregation data). Because of the increased complexity of analysis and interpretation of clinical genetic testing described in this report, the ACMG strongly recommends that clinical molecular genetic testing should be performed in a Clinical Laboratory Improvement Amendments–approved laboratory, with results interpreted by a board-certified clinical molecular geneticist or molecular genetic pathologist or the equivalent.

Genet Med advance online publication 5 March 2015

Key Words: ACMG laboratory guideline; clinical genetic testing; interpretation; reporting; sequence variant terminology; variant reporting

[1]Department of Molecular and Medical Genetics, Knight Diagnostic Laboratories, Oregon Health & Science University, Portland, Oregon, USA; [2]College of American Pathologists, Chicago, Illinois, USA; [3]GeneDx, Gaithersburg, Maryland, USA; [4]Department of Pediatrics, Section of Genetics, Medical College of Wisconsin, Milwaukee, Wisconsin, USA; [5]Department of Human Genetics, Clinical Molecular Genetics Laboratory, The University of Chicago, Chicago, Illinois, USA; [6]Cytogenetics/Molecular Genetics Laboratory, Nationwide Children's Hospital, Columbus, Ohio, USA; [7]Department of Pathology, Ohio State University College of Medicine, Columbus, Ohio, USA; [8]Department of Pediatrics, Ohio State University College of Medicine, Columbus, Ohio, USA; [9]Department of Pathology and Laboratory Medicine, University of California Los Angeles School of Medicine, Los Angeles, California, USA; [10]Department of Pediatrics, University of California Los Angeles School of Medicine, Los Angeles, California, USA; [11]Department of Human Genetics, University of California Los Angeles School of Medicine, Los Angeles, California, USA; [12]Department of Human Genetics, Emory Genetics Laboratory, Emory University, Atlanta, Georgia, USA; [13]Department of Pathology, ARUP Institute for Clinical and Experimental Pathology, University of Utah, Salt Lake City, Utah, USA; [14]Department of Pediatrics, Molecular Genetics Laboratory, Children's Hospital Colorado, University of Colorado Anschutz Medical School, Denver, Colorado, USA; [15]Partners Laboratory for Molecular Medicine and Department of Pathology, Brigham & Women's Hospital and Harvard Medical School, Boston, Massachusetts, USA; [16]Current affiliation: Phoenix Children's Hospital, Phoenix, Arizona, USA. Correspondence: Sue Richards (richarsu@ohsu.edu)

Approved by the ACMG Board of Directors on 15 December 2014 and the AMP Board of Directors on 9 January 2015.

Submitted 28 January 2015; accepted 28 January 2015; advance online publication 5 March 2015. doi:10.1038/gim.2015.30

INTRODUCTION

Clinical molecular laboratories are increasingly detecting novel sequence variants in the course of testing patient specimens for a rapidly increasing number of genes associated with genetic disorders. While some phenotypes are associated with a single gene, many are associated with multiple genes. Our understanding of the clinical significance of any given sequence variant falls along a gradient, ranging from those in which the variant is almost certainly pathogenic for a disorder to those that are almost certainly benign. While the previous American College of Medical Genetics and Genomics (ACMG) recommendations provided interpretative categories of sequence variants and an algorithm for interpretation, the recommendations did not provide defined terms or detailed variant classification guidance.[1] This report describes updated standards and guidelines for the classification of sequence variants using criteria informed by expert opinion and empirical data.

METHODS

In 2013 a workgroup consisting of ACMG, Association for Molecular Pathology (AMP), and College of American Pathologists members, representing clinical laboratory directors and clinicians, was formed with the goal of developing a recommendation for the use of standard terminology for classifying sequence variants using available evidence weighted according to a system developed through expert opinion, workgroup consensus, and community input. To assess the views of the clinical laboratory community, surveys were sent to over 100 sequencing laboratories in the United States and Canada that were listed in GeneTests.org, requesting input on terminology preferences and evaluation of evidence for classifying variants. Laboratory testing experience included rare disease as well as pharmacogenomics and somatic cancer testing. The first survey, aimed at assessing terminology preferences, was sent in February 2013, and the results were presented in an open forum at the 2013 ACMG annual meeting including over 75 attendees. Survey respondents represented more than 45 laboratories in North America. The outcome of the survey and open forum indicated that (i) a five-tier terminology system using the terms "pathogenic," "likely pathogenic," "uncertain significance," "likely benign," and "benign" was preferred and already in use by a majority of laboratories, and (ii) the first effort of the workgroup should focus on Mendelian and mitochondrial variants.

In the first survey, laboratories also were asked to provide their protocols for variant assessment, and 11 shared their methods. By analyzing all the protocols submitted, the workgroup developed a set of criteria to weight variant evidence and a set of rules for combining criteria to arrive at one of the five classification tiers. Workgroup members tested the scheme within their laboratories for several weeks using variants already classified in their laboratories and/or by the broader community. In addition, typical examples of variants harboring the most common types of evidence were tested for classification assignment to ensure the system would classify those variants according to current approaches consistently applied by workgroup members. A second survey was sent in August 2013 to the same laboratories identified through GeneTests.org as well as through AMP's listserv of ~2,000 members, along with the proposed classification scheme and a detailed supplement describing how to use each of the criteria. Laboratories were asked to use the scheme and to provide feedback as to the suitability and relative weighting of each criteria, the ease of use of the classification system, and whether they would adopt such a system in their own laboratory. Responses from over 33 laboratories indicated majority support for the proposed approach, and feedback further guided the development of the proposed standards and guidelines.

In November 2013 the workgroup held a workshop at the AMP meeting with more than 50 attendees, presenting the revised classification criteria and two potential scoring systems. One system is consistent with the approach presented here and the other is a point system whereby each criterion is given a number of points, assigning positive points for pathogenic criteria and negative points for benign criteria, with the total defining the variant class. With an audience-response system, the participants were asked how they would weight each criterion (as strong, moderate or supporting, or not used) during evaluation of variant evidence. Again, the responses were incorporated into the classification system presented here. It should be noted that while the majority of respondents did favor a point system, the workgroup felt that the assignment of specific points for each criterion implied a quantitative level of understanding of each criterion that is currently not supported scientifically and does not take into account the complexity of interpreting genetic evidence.

The workgroup also evaluated the literature for recommendations from other professional societies and working groups that have developed variant classification guidelines for well-studied genes in breast cancer, colon cancer, and cystic fibrosis and statistical analysis programs for quantitative evaluation of variants in select diseases.[2–5] While those variant analysis guidelines are useful in a specific setting, it was difficult to apply their proposed criteria to all genes and in different laboratory settings. The variant classification approach described in this article is meant to be applicable to variants in all Mendelian genes, whether identified by single gene tests, multigene panels, exome sequencing, or genome sequencing. We expect that this variant classification approach will evolve as technology and knowledge improve. We should also note that those working in specific disease groups should continue to develop more focused guidance regarding the classification of variants in specific genes given that the applicability and weight assigned to certain criteria may vary by gene and disease.

GENERAL CONSIDERATIONS

Terminology

A mutation is defined as a permanent change in the nucleotide sequence, whereas a polymorphism is defined as a variant with a frequency above 1%. The terms "mutation" and "polymorphism," however, which have been used widely,

often lead to confusion because of incorrect assumptions of pathogenic and benign effects, respectively. Thus, it is recommended that both terms be replaced by the term "variant" with the following modifiers: (i) pathogenic, (ii) likely pathogenic, (iii) uncertain significance, (iv) likely benign, or (v) benign. Although these modifiers may not address all human phenotypes, they comprise a five-tier system of classification for variants relevant to Mendelian disease as addressed in this guidance. It is recommended that all assertions of pathogenicity (including "likely pathogenic") be reported with respect to a condition and inheritance pattern (e.g., c.1521_1523delCTT (p.Phe508del), pathogenic, cystic fibrosis, autosomal recessive).

It should be noted that some laboratories may choose to have additional tiers (e.g., subclassification of variants of uncertain significance, particularly for internal use), and this practice is not considered inconsistent with these recommendations. It should also be noted that the terms recommended here differ somewhat from the current recommendations for classifying copy-number variants detected by cytogenetic microarray.[6] The schema recommended for copy-number variants, while also including five tiers, uses "uncertain clinical significance—likely pathogenic" and "uncertain clinical significance—likely benign." The majority of the workgroup was not supportive of using "uncertain significance" to modify the terms "likely pathogenic" or "likely benign" given that it was felt that the criteria presented here to classify variants into the "likely" categories included stronger evidence than outlined in the copy-number variant guideline and that combining these two categories would create confusion for the health-care providers and individuals receiving clinical reports. However, it was felt that the use of the term "likely" should be restricted to variants where the data support a high likelihood that it is pathogenic or a high likelihood that it is benign. Although there is no quantitative definition of the term "likely," guidance has been proposed in certain variant classification settings. A survey of the community during an ACMG open forum, however, suggested a much wider range of uses of the term "likely." Recognizing this, we propose that the terms "likely pathogenic" and "likely benign" be used to mean greater than 90% certainty of a variant either being disease-causing or benign to provide laboratories with a common, albeit arbitrary, definition. Similarly, the International Agency for Research on Cancer guideline[2] supports a 95% level of certainty of pathogenicity, but the workgroup (confirmed by feedback during the ACMG open forum) felt that clinicians and patients were willing to tolerate a slightly higher chance of error, leading to the 90% decision. It should also be noted that at present most variants do not have data to support a quantitative assignment of variant certainty to any of the five categories given the heterogeneous nature of most diseases. It is hoped that over time experimental and statistical approaches to objectively assign pathogenicity confidence to variants will be developed and that more rigorous approaches to defining what the clinical community desires in terms of confidence will more fully inform terminologies and likelihoods.

The use of new terminologies may require education of the community. Professional societies are encouraged to engage in educating all laboratories as well as health-care providers on the use of these terms, and laboratories also are encouraged to directly educate their ordering physicians.

Nomenclature

A uniform nomenclature, informed by a set of standardized criteria, is recommended to ensure the unambiguous designation of a variant and enable effective sharing and downstream use of genomic information. A standard gene variant nomenclature (http://www.hgvs.org/mutnomen) is maintained and versioned by the Human Genome Variation Society (HGVS),[7] and its use is recommended as the primary guideline for determining variant nomenclature except as noted.[6] Laboratories should note the version being used in their test methods. Tools are available to provide correct HGVS nomenclature for describing variants (https://mutalyzer.nl).[8] Clinical reports should include sequence reference(s) to ensure unambiguous naming of the variant at the DNA level, as well as to provide coding and protein nomenclature to assist in functional interpretations (e.g., "g." for genomic sequence, "c." for coding DNA sequence, "p." for protein, "m." for mitochondria). The coding nomenclature should be described using the "A" of the ATG translation initiation codon as position number 1. Where historical alternate nomenclature has been used, current nomenclature should be used with an additional notation of the historical naming. The reference sequence should be complete and derived from either the National Center for Biotechnology Information RefSeq database (http://www.ncbi.nlm.nih.gov/RefSeq/)[9] with the version number or the Locus Reference Genomic database (http://www.lrg-sequence.org).[10] Genomic coordinates should be used and defined according to a standard genome build (e.g., hg19) or a genomic reference sequence that covers the entire gene (including the 5′ and 3′ untranslated regions and promoter). A reference transcript for each gene should be used and provided in the report when describing coding variants. The transcript should represent either the longest known transcript and/or the most clinically relevant transcript. Community-supported reference transcripts can often be identified through Locus Reference Genomic,[10] the Consensus CDS Database,[11] the Human Gene Mutation Database (http://www.hgmd.cf.ac.uk), ClinVar (http://www.ncbi.nlm.nih.gov/clinvar), or a locus-specific database. However, laboratories should evaluate the impact of the variant on all clinically relevant transcripts, including alternate transcripts that contain additional exons or extended untranslated regions, when there are known variants in these regions that are clinically interpretable.

Not all types of variants (e.g., complex variants) are covered by the HGVS recommendations, but possible descriptions for complex variants have been reported.[7,12] In addition, this ACMG recommendation supports three specific exceptions to the HGVS nomenclature rules: (i) "X" is still considered acceptable for use in reporting nonsense variants in addition to the current HGVS recommendation of "*" and "Ter"; (ii) it is

recommended that exons be numbered according to the chosen reference transcript used to designate the variant; and (iii) the term "pathogenic" is recommended instead of "affects function" because clinical interpretation is typically directly evaluating pathogenicity.

Literature and database use

A large number of databases contain a growing number of variants that are continuously being discovered in the human genome. When classifying and reporting a variant, clinical laboratories may find valuable information in databases, as well as in the published literature. As noted above, sequence databases can also be used to identify appropriate reference sequences. Databases can be useful for gathering information but should be used with caution.

Population databases (Table 1) are useful in obtaining the frequencies of variants in large populations. Population databases cannot be assumed to include only healthy individuals and are known to contain pathogenic variants. These population databases do not contain extensive information regarding the functional effect of these variants or any possible associated phenotypes. When using population databases, one must determine whether healthy or disease cohorts were used and, if possible, whether more than one individual in a family was included, as well as the age range of the subjects.

Disease databases (Table 1) primarily contain variants found in patients with disease and assessment of the variants' pathogenicity. Disease and gene-specific databases often contain variants that are incorrectly classified, including incorrect

Table 1 Population, disease-specific, and sequence databases

Population databases	
Exome Aggregation Consortium<br>http://exac.broadinstitute.org/	Database of variants found during exome sequencing of 61,486 unrelated individuals sequenced as part of various disease-specific and population genetic studies. Pediatric disease subjects as well as related individuals were excluded.
Exome Variant Server<br>http://evs.gs.washington.edu/EVS	Database of variants found during exome sequencing of several large cohorts of individuals of European and African American ancestry. Includes coverage data to inform the absence of variation.
1000 Genomes Project<br>http://browser.1000genomes.org	Database of variants found during low-coverage and high-coverage genomic and targeted sequencing from 26 populations. Provides more diversity compared to the Exome Variant Server but also contains lower-quality data, and some cohorts contain related individuals.
dbSNP<br>http://www.ncbi.nlm.nih.gov/snp	Database of short genetic variations (typically ≤50 bp) submitted from many sources. May lack details of the originating study and may contain pathogenic variants.
dbVar<br>http://www.ncbi.nlm.nih.gov/dbvar	Database of structural variation (typically >50 bp) submitted from many sources.
Disease databases	
ClinVar<br>http://www.ncbi.nlm.nih.gov/clinvar	Database of assertions about the clinical significance and phenotype relationship of human variations.
OMIM<br>http://www.omim.org	Database of human genes and genetic conditions that also contains a representative sampling of disease-associated genetic variants.
Human Gene Mutation Database<br>http://www.hgmd.org	Database of variant annotations published in the literature. Requires fee-based subscription to access much of the content.
Locus/disease/ethnic/other-specific databases	
Human Genome Variation Society<br>http://www.hgvs.org/dblist/dblist.html	The Human Genome Variation Society site developed a list of thousands of databases that provide variant annotations on specific subsets of human variation. A large percentage of databases are built in the Leiden Open Variation Database system.
Leiden Open Variation Database<br>http://www.lovd.nl	
DECIPHER<br>http://decipher.sanger.ac.uk	A molecular cytogenetic database for clinicians and researchers linking genomic microarray data with phenotype using the Ensembl genome browser.
Sequence databases	
NCBI Genome<br>http://www.ncbi.nlm.nih.gov/genome	Source of full human genome reference sequences.
RefSeqGene<br>http://www.ncbi.nlm.nih.gov/refseq/rsg	Medically relevant gene reference sequence resource.
Locus Reference Genomic (LRG)<br>http://www.lrg-sequence.org	
MitoMap<br>http://www.mitomap.org/MITOMAP/HumanMitoSeq	Revised Cambridge reference sequence for human mitochondrial DNA.

claims published in the peer-reviewed literature, because many databases do not perform a primary review of evidence. When using disease databases, it is important to consider how patients were ascertained, as described below.

When using databases, clinical laboratories should (i) determine how frequently the database is updated, whether data curation is supported, and what methods were used for curation; (ii) confirm the use of HGVS nomenclature and determine the genome build and transcript references used for naming variants; (iii) determine the degree to which data are validated for analytical accuracy (e.g., low-pass next-generation sequencing versus Sanger-validated variants) and evaluate any quality metrics that are provided to assess data accuracy, which may require reading associated publications; and (iv) determine the source and independence of the observations listed.

Variant assessment also includes searching the scientific and medical literature. Literature using older nomenclature and classification or based on a single observation should be used with caution. When identifying individuals and families with a variant, along with associated phenotypes, it is important to consider how patients were ascertained. This caveat is important when assessing data from publications because affected individuals and related individuals are often reported multiple times, depending on the context and size of the study. This may be due to authorship overlap, interlaboratory collaborations, or a proband and family members being followed across different clinical systems. This may mistakenly lead to duplicate counting of affected patients and a false increase in variant frequency. Overlapping authorship or institutions is the first clue to the potential for overlapping data sets.

Clinical laboratories should implement an internal system to track all sequence variants identified in each gene and clinical assertions when reported. This is important for tracking genotype–phenotype correlations and the frequency of variants in affected and normal populations. Clinical laboratories are encouraged to contribute to variant databases, such as ClinVar, including clinical assertions and evidence used for the variant classification, to aid in the continued understanding of the impact of human variation. Whenever possible, clinical information should be provided following Health Insurance Portability and Accountability Act regulations for privacy. Clinical laboratories are encouraged to form collaborations with clinicians to provide clinical information to better understand how genotype influences clinical phenotype and to resolve differences in variant interpretation between laboratories. Because of the great potential to aid clinical laboratory practice, efforts are underway for clinical variant databases to be expanded and standardized. Standardization will provide easier access to updated information as well as facilitate submission from the clinical laboratory. For example, the ClinVar database allows for the deposition of variants with clinical observations and assertions, with review status tracked to enable a more transparent view of the levels of quality of the curation.

Computational (in silico) predictive programs

A variety of in silico tools, both publicly and commercially available, can aid in the interpretation of sequence variants. The algorithms used by each tool may differ but can include determination of the effect of the sequence variant at the nucleotide and amino acid level, including determination of the effect of the variant on the primary and alternative gene transcripts, other genomic elements, as well as the potential impact of the variant on the protein. The two main categories of such tools include those that predict whether a missense change is damaging to the resultant protein function or structure and those that predict whether there is an effect on splicing (**Table 2**). Newer tools are beginning to address additional noncoding sequences.[13]

The impact of a missense change depends on criteria such as the evolutionary conservation of an amino acid or nucleotide, the location and context within the protein sequence, and the biochemical consequence of the amino acid substitution. The measurement of one or a combination of these criteria is used in various in silico algorithms that assess the predicted impact of a missense change. Several efforts have evaluated the performance of available prediction software to compare them with each other and to assess their ability to predict "known" disease-causing variants.[14–17] In general, most algorithms for missense variant prediction are 65–80% accurate when examining known disease variants.[16] Most tools also tend to have low specificity, resulting in overprediction of missense changes as deleterious, and are not as reliable at predicting missense variants with a milder effect.[18] The in silico tools more commonly used for missense variant interpretation in clinical laboratories include PolyPhen2,[19] SIFT,[20] and MutationTaster.[21] A list of in silico tools used to predict missense variants can be found in **Table 2**.

Multiple software programs have been developed to predict splicing as it relates to the creation or loss of splice sites at the exonic or intronic level.[22] In general, splice site prediction tools have higher sensitivity (~90–100%) relative to specificity (~60–80%) in predicting splice site abnormalities.[23,24] Some of the in silico tools commonly used for splice site variant interpretation are listed in **Table 2**.

While many of the different software programs use different algorithms for their predictions, they have similarities in their underlying basis; therefore, predictions combined from different in silico tools are considered as a single piece of evidence in sequence interpretation as opposed to independent pieces of evidence. The use of multiple software programs for sequence variant interpretation is also recommended because the different programs each have their own strengths and weaknesses, depending on the algorithm; in many cases performance can vary by the gene and protein sequence. These are only predictions, however, and their use in sequence variant interpretation should be implemented carefully. It is not recommended that these predictions be used as the sole source of evidence to make a clinical assertion.

Table 2 In silico predictive algorithms

Category	Name	Website	Basis
Missense prediction	ConSurf	http://consurftest.tau.ac.il	Evolutionary conservation
	FATHMM	http://fathmm.biocompute.org.uk	Evolutionary conservation
	MutationAssessor	http://mutationassessor.org	Evolutionary conservation
	PANTHER	http://www.pantherdb.org/tools/csnpScoreForm.jsp	Evolutionary conservation
	PhD-SNP	http://snps.biofold.org/phd-snp/phd-snp.html	Evolutionary conservation
	SIFT	http://sift.jcvi.org	Evolutionary conservation
	SNPs&GO	http://snps-and-go.biocomp.unibo.it/snps-and-go	Protein structure/function
	Align GVGD	http://agvgd.iarc.fr/agvgd_input.php	Protein structure/function and evolutionary conservation
	MAPP	http://mendel.stanford.edu/SidowLab/downloads/MAPP/index.html	Protein structure/function and evolutionary conservation
	MutationTaster	http://www.mutationtaster.org	Protein structure/function and evolutionary conservation
	MutPred	http://mutpred.mutdb.org	Protein structure/function and evolutionary conservation
	PolyPhen-2	http://genetics.bwh.harvard.edu/pph2	Protein structure/function and evolutionary conservation
	PROVEAN	http://provean.jcvi.org/index.php	Alignment and measurement of similarity between variant sequence and protein sequence homolog
	nsSNPAnalyzer	http://snpanalyzer.uthsc.edu	Multiple sequence alignment and protein structure analysis
	Condel	http://bg.upf.edu/fannsdb/	Combines SIFT, PolyPhen-2, and MutationAssessor
	CADD	http://cadd.gs.washington.edu	Contrasts annotations of fixed/nearly fixed derived alleles in humans with simulated variants
Splice site prediction	GeneSplicer	http://www.cbcb.umd.edu/software/GeneSplicer/gene_spl.shtml	Markov models
	Human Splicing Finder	http://www.umd.be/HSF/	Position-dependent logic
	MaxEntScan	http://genes.mit.edu/burgelab/maxent/Xmaxentscan_scoreseq.html	Maximum entropy principle
	NetGene2	http://www.cbs.dtu.dk/services/NetGene2	Neural networks
	NNSplice	http://www.fruitfly.org/seq_tools/splice.html	Neural networks
	FSPLICE	http://www.softberry.com/berry.phtml?topic=fsplice&group=programs&subgroup=gfind	Species-specific predictor for splice sites based on weight matrices model
Nucleotide conservation prediction	GERP	http://mendel.stanford.edu/sidowlab/downloads/gerp/index.html	Genomic evolutionary rate profiling
	PhastCons	http://compgen.bscb.cornell.edu/phast/	Conservation scoring and identification of conserved elements
	PhyloP	http://compgen.bscb.cornell.edu/phast/	
		http://compgen.bscb.cornell.edu/phast/help-pages/phyloP.txt	Alignment and phylogenetic trees: Computation of *P* values for conservation or acceleration, either lineage-specific or across all branches

In silico tools/software prediction programs used for sequence variant interpretation.

PROPOSED CRITERIA FOR INTERPRETATION OF SEQUENCE VARIANTS

The following approach to evaluating evidence for a variant is intended for interpretation of variants observed in patients with suspected inherited (primarily Mendelian) disorders in a clinical diagnostic laboratory setting. It is not intended for the interpretation of somatic variation, pharmacogenomic (PGx) variants, or variants in genes associated with multigenic non-Mendelian complex disorders. Care must be taken when applying these rules to candidate genes ("genes of uncertain significance" (GUS)) in the context of exome or genome studies (see Special Considerations below) because this guidance is not intended to fulfill the needs of the research community in its effort to identify new genes in disease.

Although these approaches can be used for evaluating variants found in healthy individuals or secondary to the indication for testing, further caution must be used, as noted in several parts of the guideline, given the low prior likelihood that most

variants unrelated to the indication are pathogenic. Although we expect that, in general, these guidelines will apply for variant classification regardless of whether the variant was identified through analysis of a single gene, gene panel, exome, genome, or transcriptome, it is important to consider the differences between implicating a variant as pathogenic (i.e., causative) for a disease and a variant that may be predicted to be disruptive/damaging to the protein for which it codes, but is not necessarily implicated in a disease. These rules are intended to determine whether a variant in a gene with a definitive role in a Mendelian disorder may be pathogenic for that disorder. Pathogenicity determination should be independent of interpreting the cause of disease in a given patient. For example, a variant should not be reported as pathogenic in one case and not pathogenic in another simply because the variant is not thought to explain disease in a given case. Pathogenicity should be determined by the entire body of evidence in aggregate, including all cases studied, arriving at a single conclusion.

This classification approach may be somewhat more stringent than laboratories have applied to date. They may result in a larger proportion of variants being categorized as uncertain significance. It is hoped that this approach will reduce the substantial number of variants being reported as "causative" of disease without having sufficient supporting evidence for that classification. It is important to keep in mind that when a clinical laboratory reports a variant as pathogenic, health-care providers are highly likely to take that as "actionable" and to alter the treatment or surveillance of a patient[25] or remove such management in a genotype-negative family member, based on that determination (see How Should Health-Care Providers Use These Guidelines and Recommendations, below).

We have provided two sets of criteria: one for classification of pathogenic or likely pathogenic variants (**Table 3**) and one for classification of benign or likely benign variants (**Table 4**). Each pathogenic criterion is weighted as very strong (PVS1), strong (PS1–4); moderate (PM1–6), or supporting (PP1–5), and each benign criterion is weighted as stand-alone (BA1), strong (BS1–4), or supporting (BP1–6). The numbering within each category does not convey any differences of weight and is merely labeled to help refer to the different criteria. For a given variant, the user selects the criteria based on the evidence observed for the variant. The criteria then are combined according to the scoring rules in **Table 5** to choose a classification from the five-tier system. The rules apply to all available data on a variant, whether gathered from the current case under investigation or from well-vetted previously published data. Unpublished case data may also be obtained through public resources (e.g., ClinVar or locus specific databases) and from a laboratory's own database. To provide critical flexibility to variant classification, some criteria listed as one weight can be moved to another weight using professional judgment, depending on the evidence collected. For example, rule PM3 could be upgraded to strong if there were multiple observations of detection of the variant in *trans* (on opposite chromosomes) with other pathogenic variants (see PM3 BP2 cis/trans Testing for further guidance). By contrast, in situations when the data are not as strong as described, judgment can be used to consider the evidence as fulfilling a lower level (e.g., see PS4, Note 2 in **Table 3**). If a variant does not fulfill criteria using either of these sets (pathogenic or benign), or the evidence for benign and pathogenic is conflicting, the variant defaults to uncertain significance. The criteria, organized by type and strength, is shown in **Figure 1**. Please note that expert judgment must be applied when evaluating the full body of evidence to account for differences in the strength of variant evidence.

The following is provided to more thoroughly explain certain concepts noted in the criteria for variant classification (**Tables 3** and **4**) and to provide examples and/or caveats or pitfalls in their use. This section should be read in concert with **Tables 3** and **4**.

PVS1 null variants

Certain types of variants (e.g., nonsense, frameshift, canonical ±1 or 2 splice sites, initiation codon, single exon or multiexon deletion) can often be assumed to disrupt gene function by leading to a complete absence of the gene product by lack of transcription or nonsense-mediated decay of an altered transcript. One must, however, exercise caution when classifying these variants as pathogenic by considering the following principles:

(i) When classifying such variants as pathogenic, one must ensure that null variants are a known mechanism of pathogenicity consistent with the established inheritance pattern for the disease. For example, there are genes for which only heterozygous missense variants cause disease and null variants are benign in a heterozygous state (e.g., many hypertrophic cardiomyopathy genes). A novel heterozygous nonsense variant in the *MYH7* gene would not be considered pathogenic for dominant hypertrophic cardiomyopathy based solely on this evidence, whereas a novel heterozygous nonsense variant in the *CFTR* gene would likely be considered a recessive pathogenic variant.

(ii) One must also be cautious when interpreting truncating variants downstream of the most 3′ truncating variant established as pathogenic in the literature. This is especially true if the predicted stop codon occurs in the last exon or in the last 50 base pairs of the penultimate exon, such that nonsense-mediated decay[26] would not be predicted, and there is a higher likelihood of an expressed protein. The length of the predicted truncated protein would also factor into the pathogenicity assignment, however, and such variants cannot be interpreted without a functional assay.

(iii) For splice-site variants, the variant may lead to exon skipping, shortening, or inclusion of intronic material as a result of alternative donor/acceptor site usage or creation of new sites. Although splice-site variants are predicted to lead to a null effect, confirmation of impact requires functional analysis by either RNA or protein analysis. One must also consider the possibility of an in-frame deletion/insertion, which could retain the critical domains of the protein and hence lead to

Table 3 Criteria for classifying pathogenic variants

Evidence of pathogenicity	Category
Very strong	PVS1 null variant (nonsense, frameshift, canonical ±1 or 2 splice sites, initiation codon, single or multiexon deletion) in a gene where LOF is a known mechanism of disease<br>Caveats:<br>• Beware of genes where LOF is not a known disease mechanism (e.g., *GFAP*, *MYH7*)<br>• Use caution interpreting LOF variants at the extreme 3′ end of a gene<br>• Use caution with splice variants that are predicted to lead to exon skipping but leave the remainder of the protein intact<br>• Use caution in the presence of multiple transcripts
Strong	PS1 Same amino acid change as a previously established pathogenic variant regardless of nucleotide change<br>Example: Val→Leu caused by either G>C or G>T in the same codon<br>Caveat: Beware of changes that impact splicing rather than at the amino acid/protein level<br>PS2 De novo (both maternity and paternity confirmed) in a patient with the disease and no family history<br>Note: Confirmation of paternity only is insufficient. Egg donation, surrogate motherhood, errors in embryo transfer, and so on, can contribute to nonmaternity.<br>PS3 Well-established in vitro or in vivo functional studies supportive of a damaging effect on the gene or gene product<br>Note: Functional studies that have been validated and shown to be reproducible and robust in a clinical diagnostic laboratory setting are considered the most well established.<br>PS4 The prevalence of the variant in affected individuals is significantly increased compared with the prevalence in controls<br>Note 1: Relative risk or OR, as obtained from case–control studies, is >5.0, and the confidence interval around the estimate of relative risk or OR does not include 1.0. See the article for detailed guidance.<br>Note 2: In instances of very rare variants where case–control studies may not reach statistical significance, the prior observation of the variant in multiple unrelated patients with the same phenotype, and its absence in controls, may be used as moderate level of evidence.
Moderate	PM1 Located in a mutational hot spot and/or critical and well-established functional domain (e.g., active site of an enzyme) without benign variation<br>PM2 Absent from controls (or at extremely low frequency if recessive) (**Table 6**) in Exome Sequencing Project, 1000 Genomes Project, or Exome Aggregation Consortium<br>Caveat: Population data for insertions/deletions may be poorly called by next-generation sequencing.<br>PM3 For recessive disorders, detected in *trans* with a pathogenic variant<br>Note: This requires testing of parents (or offspring) to determine phase.<br>PM4 Protein length changes as a result of in-frame deletions/insertions in a nonrepeat region or stop-loss variants<br>PM5 Novel missense change at an amino acid residue where a different missense change determined to be pathogenic has been seen before<br>Example: Arg156His is pathogenic; now you observe Arg156Cys<br>Caveat: Beware of changes that impact splicing rather than at the amino acid/protein level.<br>PM6 Assumed de novo, but without confirmation of paternity and maternity
Supporting	PP1 Cosegregation with disease in multiple affected family members in a gene definitively known to cause the disease<br>Note: May be used as stronger evidence with increasing segregation data<br>PP2 Missense variant in a gene that has a low rate of benign missense variation and in which missense variants are a common mechanism of disease<br>PP3 Multiple lines of computational evidence support a deleterious effect on the gene or gene product (conservation, evolutionary, splicing impact, etc.)<br>Caveat: Because many in silico algorithms use the same or very similar input for their predictions, each algorithm should not be counted as an independent criterion. PP3 can be used only once in any evaluation of a variant.<br>PP4 Patient’s phenotype or family history is highly specific for a disease with a single genetic etiology<br>PP5 Reputable source recently reports variant as pathogenic, but the evidence is not available to the laboratory to perform an independent evaluation

LOF, loss of function; OR, odds ratio.

Table 4 Criteria for classifying benign variants

Evidence of benign impact	Category
Stand-alone	BA1 Allele frequency is >5% in Exome Sequencing Project, 1000 Genomes Project, or Exome Aggregation Consortium
Strong	BS1 Allele frequency is greater than expected for disorder (see Table 6)
	BS2 Observed in a healthy adult individual for a recessive (homozygous), dominant (heterozygous), or X-linked (hemizygous) disorder, with full penetrance expected at an early age
	BS3 Well-established in vitro or in vivo functional studies show no damaging effect on protein function or splicing
	BS4 Lack of segregation in affected members of a family
	Caveat: The presence of phenocopies for common phenotypes (i.e., cancer, epilepsy) can mimic lack of segregation among affected individuals. Also, families may have more than one pathogenic variant contributing to an autosomal dominant disorder, further confounding an apparent lack of segregation.
Supporting	BP1 Missense variant in a gene for which primarily truncating variants are known to cause disease
	BP2 Observed in *trans* with a pathogenic variant for a fully penetrant dominant gene/disorder or observed in *cis* with a pathogenic variant in any inheritance pattern
	BP3 In-frame deletions/insertions in a repetitive region without a known function
	BP4 Multiple lines of computational evidence suggest no impact on gene or gene product (conservation, evolutionary, splicing impact, etc.)
	Caveat: Because many in silico algorithms use the same or very similar input for their predictions, each algorithm cannot be counted as an independent criterion. BP4 can be used only once in any evaluation of a variant.
	BP5 Variant found in a case with an alternate molecular basis for disease
	BP6 Reputable source recently reports variant as benign, but the evidence is not available to the laboratory to perform an independent evaluation
	BP7 A synonymous (silent) variant for which splicing prediction algorithms predict no impact to the splice consensus sequence nor the creation of a new splice site AND the nucleotide is not highly conserved

either a mild or neutral effect with a minor length change (PM4) or a gain-of-function effect.

(iv) Considering the presence of alternate gene transcripts and understanding which are biologically relevant, and in which tissues the products are expressed, are important. If a truncating variant is confined to only one or not all transcripts, one must be cautious about overinterpreting variant impact given the presence of the other protein isoforms.

(v) One must also be cautious in assuming that a null variant will lead to disease if found in an exon where no other pathogenic variants have been described, given the possibility that the exon may be alternatively spliced. This is particularly true if the predicted truncating variant is identified as an incidental finding (unrelated to the indication for testing), given the low prior likelihood of finding a pathogenic variant in that setting.

PS1 same amino acid change

In most cases, when one missense variant is known to be pathogenic, a different nucleotide change that results in the same amino acid (e.g., c.34G>C (p.Val12Leu) and c.34G>T (p.Val12Leu)) can also be assumed to be pathogenic, particularly if the mechanism of pathogenicity occurs through altered protein function. However, it is important to assess the possibility that the variant may act directly through the specific DNA change (e.g., through splicing disruption as assessed by at least computational analysis) instead of through the amino acid change, in which case the assumption of pathogenicity may no longer be valid.

PS2 PM6 de novo variants

A variant observed to have arisen de novo (parental samples testing negative) is considered strong support for pathogenicity if the following conditions are met:

(i) Both parental samples were shown through identity testing to be from the biological parents of the patient. Note that PM6 applies if identity is assumed but not confirmed.

(ii) The patient has a family history of disease that is consistent with de novo inheritance (e.g., unaffected parents for a dominant disorder). It is possible, however, that more than one sibling may be affected because of germ-line mosaicism.

(iii) The phenotype in the patient matches the gene's disease association with reasonable specificity. For example, this argument is strong for a patient with a de novo variant in the *NIPBL* gene who has distinctive facial features, hirsutism, and upper-limb defects (i.e., Cornelia de Lange syndrome), whereas it would be weaker for a de novo variant found by exome sequencing in a child with nonspecific features such as developmental delay.

PS3 BS3 functional studies

Functional studies can be a powerful tool in support of pathogenicity; however, not all functional studies are effective in

predicting an impact on a gene or protein function. For example, certain enzymatic assays offer well-established approaches to assess the impact of a missense variant on enzymatic function in a metabolic pathway (e.g., α-galactosidase enzyme function). On the other hand, some functional assays may be less consistent predictors of the effect of variants on protein function. To assess the validity of a functional assay, one must consider how closely the functional assay reflects the biological environment. For example, assaying enzymatic function directly from biopsied tissue from the patient or an animal model provides stronger evidence than expressing the protein in vitro. Likewise, evidence is stronger if the assay reflects the full biological function of the protein (e.g., substrate breakdown by an enzyme) compared with only one component of function (e.g., adenosine triphosphate hydrolysis for a protein with additional binding properties). Validation, reproducibility, and robustness data that assess the analytical performance of the assay and account for specimen integrity, which can be affected by the method and time of acquisition, as well as storage and transport, are important factors to consider. These factors are mitigated in the case of an assay in a Clinical Laboratory Improvement Amendments laboratory–developed test or commercially available kit. Assays that assess the impact of variants at the messenger RNA level can be highly informative when evaluating the effects of variants at splice junctions and within coding sequences and untranslated regions, as well as deeper intronic regions (e.g., messenger RNA stability, processing, or translation). Technical approaches include direct analysis of RNA and/or complementary DNA derivatives and in vitro minigene splicing assays.

PS4 PM2 BA1 BS1 BS2 variant frequency and use of control populations

Assessing the frequency of a variant in a control or general population is useful in assessing its potential pathogenicity. This can be accomplished by searching publicly available population databases (e.g., 1000 Genomes Project, National Heart, Lung, and Blood Institute Exome Sequencing Project Exome Variant Server, Exome Aggregation Consortium; **Table 1**), as well as using race-matched control data that often are published in the literature. The Exome Sequencing Project data set is useful for Caucasian and African American populations and has coverage data to determine whether a variant is absent. Although the 1000 Genomes Project data cannot be used to assess the absence of a variant, it has a broader representation of different racial populations. The Exome Aggregation Consortium more recently released allele frequency data from >60,000 exomes from a diverse set of populations that includes approximately two-thirds of the Exome Sequencing Project data. In general, an allele frequency in a control population that is greater than expected for the disorder (**Table 6**) is considered strong support for a benign interpretation for a rare Mendelian disorder (BS1) or, if over 5%, it is considered as stand-alone support (BA1). Furthermore, if the disease under investigation is

Table 5 Rules for combining criteria to classify sequence variants

Pathogenic	(i) 1 Very strong (PVS1) *AND*
	(a) ≥1 Strong (PS1–PS4) *OR*
	(b) ≥2 Moderate (PM1–PM6) *OR*
	(c) 1 Moderate (PM1–PM6) and 1 supporting (PP1–PP5) *OR*
	(d) ≥2 Supporting (PP1–PP5)
	(ii) ≥2 Strong (PS1–PS4) *OR*
	(iii) 1 Strong (PS1–PS4) *AND*
	(a)≥3 Moderate (PM1–PM6) *OR*
	(b)2 Moderate (PM1–PM6) *AND* ≥2 Supporting (PP1–PP5) *OR*
	(c)1 Moderate (PM1–PM6) *AND* ≥4 supporting (PP1–PP5)
Likely pathogenic	(i) 1 Very strong (PVS1) *AND* 1 moderate (PM1–PM6) *OR*
	(ii) 1 Strong (PS1–PS4) *AND* 1–2 moderate (PM1–PM6) *OR*
	(iii) 1 Strong (PS1–PS4) *AND* ≥2 supporting (PP1–PP5) *OR*
	(iv) ≥3 Moderate (PM1–PM6) *OR*
	(v) 2 Moderate (PM1–PM6) *AND* ≥2 supporting (PP1–PP5) *OR*
	(vi) 1 Moderate (PM1–PM6) *AND* ≥4 supporting (PP1–PP5)
Benign	(i) 1 Stand-alone (BA1) *OR*
	(ii) ≥2 Strong (BS1–BS4)
Likely benign	(i) 1 Strong (BS1–BS4) and 1 supporting (BP1–BP7) *OR*
	(ii) ≥2 Supporting (BP1–BP7)
Uncertain significance	(i) Other criteria shown above are not met OR
	(ii) the criteria for benign and pathogenic are contradictory

fully penetrant at an early age and the variant is observed in a well-documented healthy adult individual for a recessive (homozygous), dominant (heterozygous), or X-linked (hemizygous) condition, then this is considered strong evidence for a benign interpretation (BS2). If the variant is absent, one should confirm that the read depth in the database is sufficient for an accurate call at the variant site. If a variant is absent from (or below the expected carrier frequency if recessive) a large general population or a control cohort (>1,000 individuals) and the population is race-matched to the patient harboring the identified variant, then this observation can be considered a moderate piece of evidence for pathogenicity (PM2). Many benign variants are "private" (unique to individuals or families), however, and therefore absence in a race-matched population is not considered sufficient or even strong evidence for pathogenicity.

The use of population data for case–control comparisons is most useful when the populations are well phenotyped, have

	Benign		Pathogenic			
	**Strong**	**Supporting**	**Supporting**	**Moderate**	**Strong**	**Very strong**
**Population data**	MAF is too high for disorder BA1/BS1 **OR** observation in controls inconsistent with disease penetrance BS2			Absent in population databases PM2	Prevalence in affecteds statistically increased over controls PS4	
**Computational and predictive data**		Multiple lines of computational evidence suggest no impact on gene /gene product BP4<br><br>Missense in gene where only truncating cause disease BP1<br><br>Silent variant with non predicted splice impact BP7<br><br>In-frame indels in repeat w/out known function BP3	Multiple lines of computational evidence support a deleterious effect on the gene /gene product PP3	Novel missense change at an amino acid residue where a different pathogenic missense change has been seen before PM5<br><br>Protein length changing variant PM4	Same amino acid change as an established pathogenic variant PS1	Predicted null variant in a gene where LOF is a known mechanism of disease PVS1
**Functional data**	Well-established functional studies show no deleterious effect BS3		Missense in gene with low rate of benign missense variants and path. missenses common PP2	Mutational hot spot or well-studied functional domain without benign variation PM1	Well-established functional studies show a deleterious effect PS3	
**Segregation data**	Nonsegregation with disease BS4		Cosegregation with disease in multiple affected family members PP1	Increased segregation data →	→	
**De novo data**				De novo (without paternity & maternity confirmed) PM6	De novo (paternity and maternity confirmed) PS2	
**Allelic data**		Observed in *trans* with a dominant variant BP2<br><br>Observed in *cis* with a pathogenic variant BP2		For recessive disorders, detected in trans with a pathogenic variant PM3		
**Other database**		Reputable source w/out shared data = benign BP6	Reputable source = pathogenic PP5			
**Other data**		Found in case with an alternate cause BP5	Patient's phenotype or FH highly specific for gene PP4			

Figure 1 Evidence framework. This chart organizes each of the criteria by the type of evidence as well as the strength of the criteria for a benign (left side) or pathogenic (right side) assertion. Evidence code descriptions can be found in **Tables 3** and **4**. BS, benign strong; BP, benign supporting; FH, family history; LOF, loss of function; MAF, minor allele frequency; path., pathogenic; PM, pathogenic moderate; PP, pathogenic supporting; PS, pathogenic strong; PVS, pathogenic very strong.

large frequency differences, and the Mendelian disease under study is early onset. Patients referred to a clinical laboratory for testing are likely to include individuals sent to "rule out" a disorder, and thus they may not qualify as well-phenotyped cases. When using a general population as a control cohort, the presence of individuals with subclinical disease is always a possibility. In both of these scenarios, however, a case–control comparison will be underpowered with respect to detecting a difference; as such, showing a statistically significant difference can still be assumed to provide supportive evidence for pathogenicity, as noted above. By contrast, the absence of a statistical difference, particularly with extremely rare variants and less penetrant phenotypes, should be interpreted cautiously.

Odds ratios (ORs) or relative risk is a measure of association between a genotype (i.e., the variant is present in the genome) and a phenotype (i.e., affected with the disease/outcome) and can be used for either Mendelian diseases or complex traits. In this guideline we are addressing only its use in Mendelian disease. While relative risk is different from the OR, relative risk asymptotically approaches ORs for small probabilities. An OR of 1.0 means that the variant does not affect the odds of having the disease, values above 1.0 mean there is an association between the variant and the risk of disease, and those below 1.0 mean there is a negative association between the variant and the risk of disease. In general, variants with a modest Mendelian effect size will have an OR of 3 or greater, whereas highly penetrant variants will have very high ORs; for example, *APOE* E4/E4 homozygotes compared with E3/E3 homozygotes have an OR of 13 (https://www.tgen.org/home/education-outreach/past-summer-interns/2012-summer-interns/erika-kollitz.aspx#.VOSi3C7G_vY). However, the confidence interval (CI) around the OR is as important as the measure of association itself. If the CI includes 1.0 (e.g., OR = 2.5, CI = 0.9–7.4), there is little confidence in

Table 6 Assessment of variant frequency in the general population for curated variant classification

Disease	Gene	Inheritance	Population	Incidence	Carrier frequency	Common mutation	Variant classification	ESP6500 AA MAF	ESP6500 EA MAF	ESP6500 All MAF	Con-cordance	Criteria to support classification
Cystic fibrosis	CFTR	AR	Caucasian	0.031%	3.6%	p.F508del	Ex24:p.F508del (Pathogenic)	N/A	N/A	N/A	N/A	ref. 57 (note variant not available in EVS)
							Ex11:c.1523T>G / p.F508C (Benign)	0.070%	0.150%	0.120%	No	ref. 58
							Ex23:c.3870A>G / p.(=) (Benign)	15.090%	2.970%	7.070%	Partial	AA MAF
							5′ UTR:c.-8G>C (Benign)	1.160%	5.550%	4.060%	Partial	EA MAF
							IVS6:c.743+40A>G (Benign)	0.700%	5.190%	3.670%	Partial	EA MAF
Phenylketoneuria	PAH	AR	North European	0.010%	2.0%		Ex12:c.1242C>T / p.(=) (Benign)	0.360%	1.310%	0.990%	No	PAH database (http://www.pahdb.mcgill.ca/)
							Ex12:c.1278T>C / p.(=) (Benign)	13.550%	0.090%	4.650%	Partial	AA MAF
							IVS12:c.1316-35C>T (Benign)	0.320%	2.630%	1.850%	Partial	EA MAF
							Ex9:c.963C>T / p.(=) (Benign)	5.170%	0.000%	1.750%	Partial	AA MAF
MCADD	ACADM	AR	Not specific	0.006%	1.5%	p.K329E aka p.K304E	Ex7:c.489T>G / p.(=) (Benign)	7.010%	0.050%	2.410%	Partial	AA MAF
ARPKD	PKHD1	AR	Not specific	0.005%	1.4%		IVS20:c.1964+17G>T (Benign)	0.200%	0.810%	0.610%	No	AA MAF
							Ex61:c.10515C>A / p.S3505R (Benign)	0.230%	1.130%	0.820%	No	EA MAF
							Ex66:c.11738G>A / p.R3913H (Benign)	1.270%	0.000%	0.430%	No	AA MAF
							Ex17:c.1587T>C / p.(=) (Benign)	1.380%	6.860%	5.010%	Partial	EA MAF
							Ex65:c.11525G>T / p.R3842L (Benign)	0.360%	2.430%	1.730%	Partial	EA MAF
							Ex61:c.10585G>C / p.E3529Q (Benign)	3.950%	0.010%	1.350%	Partial	AA MAF
Rett syndrome	MECP2	X Linked	Not specific	0.012%	De novo		Ex4:c.1161C>T / p.(=) (Benign)	0.030%	0.000%	0.010%	Partial	AA MAF
							Ex4:c.608C>T / p.T203M (Benign)	0.000%	0.060%	0.040%	Partial	http://www.ncbi.nlm.nih.gov/clinvar/variation/95198/
							Ex4:c.683C>G / p.T228S (Pathogenic)	0.830%	0.000%	0.300%	Partial	RETT database
Kabuki syndrome	KMT2D (MLL2)	AD	Not specific	0.003%	De novo		IVS31:c.8047-15C>T (Benign)	0.000%	0.020%	0.020%	Partial	EA MAF
							Ex31:c.6836G>A / p.Gly2279E (Benign)	0.000%	0.120%	0.080%	Partial	EA MAF
CHARGE syndrome	CHD7	AD	Not specific	0.010%	De novo		Ex2:c.309G>A / p.(=) (Benign)	1.460%	0.000%	0.490%	Partial	AA MAF
							Ex31:c.6478G>A / p.A2160T (Benign)	1.250%	0.000%	0.390%	Partial	AA MAF
							Ex2:c.856A>G / p.R286Gly (Benign)	0.780%	0.000%	0.250%	Partial	AA MAF
GJB2 associated hearing loss	GJB2	AR	Not specific	0.067%	2.5%	c.35delG	Ex2:c.35delG (Pathogenic)	0.090%	1.080%	0.740%	No	ref. 59
Hemochromatosis	HFE	AR	All	0.040%	8.3%	p.C282Y	Ex4:c.845G>A / p.C282Y (Other Reportable)	1.520%	6.410%	4.750%	No	http://www.ncbi.nlm.nih.gov/sites/GeneTests/review/gene/HFE

Variants in some common genetic disorders with their known incidences for dominant (Kabuki syndrome and CHARGE syndrome), X-linked diseases (Rett syndrome), and carrier frequencies for recessive disorders (cystic fibrosis, phenylketonuria (PKU), medium co-acyl dehydrogenase deficiency (MCADD), autosomal recessive polycystic kidney disease (ARPKD), GJB2-associated hearing loss, and hemochromatosis) are shown. Some variants in these genes found in the Exome Sequencing Project-GO database are listed with the variant classifications based on evidence and concordance with allele frequencies in African American (AA) and European American (EA) subpopulations. Variants that have an allele frequency greater than expected for the disorder in at least one subpopulation (AA or EA) of individuals not known to have the disorder can be considered to have strong evidence of benign impact (BS1). Variants known to be pathogenic for dominant disorders should have allele frequencies in the general population below the population disease incidence, and pathogenic variants for recessive disorders should have heterozygous frequencies consistent with their disease incidence. The allele frequencies shown for certain variants are lower for certain benign and higher for certain pathogenic variants than the disease incidence/carrier frequency; therefore, other data are required to classify these variants. Variants represented in the concordance column are designated as partial when a subpopulation frequency does not conform as above or when concordance is not achieved. Variants are designated as having partial concordance when a single subpopulation frequency is inconsistent with the classification.

the assertion of association. In the above *APOE* example the CI was ~10–16. Very simple OR calculators are available on the Internet (e.g., http://www.hutchon.net/ConfidOR.htm/ and http://easycalculation.com/statistics/odds-ratio.php/).[27,28]

PM1 mutational hot spot and/or critical and well-established functional domain

Certain protein domains are known to be critical to protein function, and all missense variants in these domains identified to date have been shown to be pathogenic. These domains must also lack benign variants. In addition, mutational hotspots in less well-characterized regions of genes are reported, in which pathogenic variants in one or several nearby residues have been observed with greater frequency. Either evidence can be considered moderate evidence of pathogenicity.

PM3 BP2 *cis/trans* testing

Testing parental samples to determine whether the variant occurs in *cis* (the same copy of the gene) or in *trans* (different copies of the gene) can be important for assessing pathogenicity. For example, when two heterozygous variants are identified in a gene for a recessive disorder, if one variant is known to be pathogenic, then determining that the other variant is in *trans* can be considered moderate evidence for pathogenicity of the latter variant (PM3). In addition, this evidence could be upgraded to strong if there are multiple observations of the variant in *trans* with other pathogenic variants. If the variant is present among the general population, however, a statistical approach would be needed to control for random co-occurrence. By contrast, finding the second variant in *cis* would be supporting, though not definitive, evidence for a benign role (BP2). In the case of uncertain pathogenicity of two heterozygous variants identified in a recessive gene, then the determination of the *cis* versus *trans* nature of the variants does not necessarily provide additional information with regard to the pathogenicity of either variant. However, the likelihood that both copies of the gene are impacted is reduced if the variants are found in *cis*.

In the context of dominant disorders the detection of a variant in *trans* with a pathogenic variant can be considered supporting evidence for a benign impact (BP2) or, in certain well-developed disease models, may even be considered stand-alone evidence, as has been validated for use in assessing *CFTR* variants.[3]

PM4 BP3 protein length changes due to in-frame deletions/insertions and stop losses

The deletion or insertion of one or more amino acids as well as the extension of a protein by changing the stop codon to an amino acid codon (e.g., a stop loss variant) is more likely to disrupt protein function compared with a missense change alone as a result of length changes in the protein. Therefore, in-frame deletions/insertions and stop losses are considered moderate evidence of pathogenicity. The larger the deletion, insertion, or extension, and the more conserved the amino acids are in a deleted region, the more substantial is the evidence to support pathogenicity. By contrast, small in-frame deletions/insertions in repetitive regions, or regions that are not well conserved in evolution, are less likely to be pathogenic.

PM5 novel missense at the same position

A novel missense amino acid change occurring at the same position as another pathogenic missense change (e.g., Trp38Ser and Trp38Leu) is considered moderate evidence but cannot be assumed to be pathogenic. This is especially true if the novel change is more conservative compared with the established pathogenic missense variant. Also, the different amino acid change could lead to a different phenotype. For example, different substitutions of the Lys650 residue of the *FGFR3* gene are associated with a wide range of clinical phenotypes: p.Lys650Gln or p.Lys650Asn causes mild hypochondroplasia; p.Lys650Met causes severe achondroplasia with developmental delay and acanthosis nigricans; and thanatophoric dysplasia type 2, a lethal skeletal dysplasia, arises from p.Lys650Glu.

PP1 BS4 segregation analysis

Care must be taken when using segregation of a variant in a family as evidence for pathogenicity. In fact, segregation of a particular variant with a phenotype in a family is evidence for linkage of the locus to the disorder but not evidence of the pathogenicity of the variant itself. A statistical approach has been published[29,30] with the caveat that the identified variant may be in linkage disequilibrium with the true pathogenic variant in that family. Statistical modeling takes into account age-related penetrance and phenocopy rates, with advanced methods also incorporating in silico predictions and co-occurrence with a known pathogenic variant into a single quantitative measure of pathogenicity.[31] Distant relatives are important to include because they are less likely to have both the disease and the variant by chance than members within a nuclear family. Full gene sequencing (including entire introns and 5′ and 3′ untranslated regions) may provide greater evidence that another variant is not involved or identify additional variants to consider as possibly causative. Unless the genetic locus is evaluated carefully, one risks misclassifying a nonpathogenic variant as pathogenic.

When a specific variant in the target gene segregates with a phenotype or disease in multiple affected family members and multiple families from diverse ethnic backgrounds, linkage disequilibrium and ascertainment bias are less likely to confound the evidence for pathogenicity. In this case, this criterion may be taken as moderate or strong evidence, depending on the extent of segregation, rather than supporting evidence.

On the other hand, lack of segregation of a variant with a phenotype provides strong evidence against pathogenicity. Careful clinical evaluation is needed to rule out mild symptoms of reportedly unaffected individuals, as well as possible phenocopies (affected individuals with disease due to a nongenetic or different genetic cause). Also, biological family relationships need to be confirmed to rule out adoption, nonpaternity,

sperm and egg donation, and other nonbiological relationships. Decreased and age-dependent penetrance also must be considered to ensure that asymptomatic family members are truly unaffected.

Statistical evaluation of cosegregation may be difficult in the clinical laboratory setting. If appropriate families are identified, clinical laboratories are encouraged to work with experts in statistical or population genetics to ensure proper modeling and to avoid incorrect conclusions of the relevance of the variant to the disease.

PP2 BP1 variant spectrum

Many genes have a defined spectrum of pathogenic and benign variation. For genes in which missense variation is a common cause of disease and there is very little benign variation in the gene, a novel missense variant can be considered supporting evidence for pathogenicity (PP2). By contrast, for genes in which truncating variants are the only known mechanism of variant pathogenicity, missense variants can be considered supporting evidence for a benign impact (BP1). For example, truncating variants in *ASPM* are the primary type of pathogenic variant in this gene, which causes autosomal recessive primary microcephaly, and the gene has a high rate of missense polymorphic variants. Therefore missense variants in *ASPM* can be considered to have this line of supporting evidence for a benign impact.

PP3 BP4 computational (in silico) data

Not overestimating computational evidence is important, particularly given that different algorithms may rely on the same (or similar) data to support predictions and most algorithms have not been validated against well-established pathogenic variants. In addition, algorithms can have vastly different predictive capabilities for different genes. If all of the in silico programs tested agree on the prediction, then this evidence can be counted as supporting. If in silico predictions disagree, however, then this evidence should not be used in classifying a variant. The variant amino acid change being present in multiple nonhuman mammalian species in an otherwise well-conserved region, suggesting the amino acid change would not compromise function, can be considered strong evidence for a benign interpretation. One must, however, be cautious about assuming a benign impact in a nonconserved region if the gene has recently evolved in humans (e.g., genes involved in immune function).

PP4 using phenotype to support variant claims

In general, the fact that a patient has a phenotype that matches the known spectrum of clinical features for a gene is not considered evidence for pathogenicity given that nearly all patients undergoing disease-targeted tests have the phenotype in question. If the following criteria are met, however, the patient's phenotype can be considered supporting evidence: (i) the clinical sensitivity of testing is high, with most patients testing positive for a pathogenic variant in that gene; (ii) the patient has a well-defined syndrome with little overlap with other clinical presentations (e.g., Gorlin syndrome including basal cell carcinoma, palmoplantar pits, odontogenic keratocysts); (iii) the gene is not subject to substantial benign variation, which can be determined through large general population cohorts (e.g., Exome Sequencing Project); and (iv) family history is consistent with the mode of inheritance of the disorder.

PP5 BP6 reputable source

There are increasing examples where pathogenicity classifications from a reputable source (e.g., a clinical laboratory with long-standing expertise in the disease area) have been shared in databases, yet the evidence that formed the basis for classification was not provided and may not be easily obtainable. In this case, the classification, if recently submitted, can be used as a single piece of supporting evidence. However, laboratories are encouraged to share the basis for classification as well as communicate with submitters to enable the underlying evidence to be evaluated and built upon. If the evidence is available, this criterion should not be used; instead, the criteria relevant to the evidence should be used.

BP5 alternate locus observations

When a variant is observed in a case with a clear alternate genetic cause of disease, this is generally considered supporting evidence to classify the variant as benign. However, there are exceptions. An individual can be a carrier of an unrelated pathogenic variant for a recessive disorder; therefore, this evidence is much stronger support for a likely benign variant classification in a gene for a dominant disorder compared with a gene for a recessive disorder. In addition, there are disorders in which having multiple variants can contribute to more severe disease. For example, two variants, one pathogenic and one novel, are identified in a patient with a severe presentation of a dominant disease. A parent also has mild disease. In this case, one must consider the possibility that the novel variant could also be pathogenic and contributing to the increased severity of disease in the proband. In this clinical scenario, observing the novel variant as the second variant would not support a benign classification of the novel variant (though it is also not considered support for a pathogenic classification without further evidence). Finally, there are certain diseases in which multigenic inheritance is known to occur, such as Bardet-Beidel syndrome, in which case the additional variant in the second locus may also be pathogenic but should be reported with caution.

BP7 synonymous variants

There is increasing recognition that splicing defects, beyond disruption of the splice consensus sequence, can be an important mechanism of pathogenicity, particularly for genes in which loss of function is a common mechanism of disease. Therefore, one should be cautious in assuming that a synonymous nucleotide change will have no effect. However, if the nucleotide position is not conserved over evolution and splicing assessment algorithms predict neither an impact to a splice consensus sequence nor the creation of a new alternate splice consensus sequence, then a splicing impact is less likely. Therefore, if

supported by computational evidence (BP4), one can classify novel synonymous variants as likely benign. However, if computational evidence suggests a possible impact on splicing or there is raised suspicion for an impact (e.g., the variant occurs in *trans* with a known pathogenic variant in a gene for a recessive disorder), then the variant should be classified as uncertain significance until a functional evaluation can provide a more definitive assessment of impact or other evidence is provided to rule out a pathogenic role.

REPORTING SEQUENCE VARIANTS

Writing succinct yet informative clinical reports can be a challenge as the complexity of the content grows from reporting variants in single genes to multigene panels to exomes and genomes. Several guidance documents have been developed for reporting, including full sample reports of the ACMG clinical laboratory standards for next-generation sequencing guidance.[32–35] Clinical reports are the final product of laboratory testing and often are integrated into a patient's electronic health record. Therefore, effective reports are concise, yet easy to understand. Reports should be written in clear language that avoids medical genetics jargon or defines such terms when used. The report should contain all of the essential elements of the test performed, including structured results, an interpretation, references, methodology, and appropriate disclaimers. These essential elements of the report also are emphasized by Clinical Laboratory Improvement Amendments regulations and the College of American Pathologists laboratory standards for next-generation sequencing clinical tests.[36]

Results

The results section should list variants using HGVS nomenclature (see Nomenclature). Given the increasing number of variants found in genetic tests, presenting the variants in tabular form with essential components may best convey the information. These components include nomenclature at both the nucleotide (genomic and complementary DNA) and protein level, gene name, disease, inheritance, exon, zygosity, and variant classification. An example of a table to report structured elements of a variant is found in the **Supplementary Appendix S1** online. Parental origin may also be included if known. In addition, if specific variants are analyzed in a genotyping test, the laboratory should specifically note the variants interrogated, with their full description and historical nomenclature if it exists. Furthermore, when reporting results from exome or genome sequencing, or occasionally very large disease-targeted panels, grouping variants into categories such as "Variants in Disease Genes with an Established Association with the Reported Phenotype," "Variants in Disease Genes with a Likely Association with the Reported Phenotype," and (where appropriate) "Incidental (Secondary) Findings" may be beneficial.

Interpretation

The interpretation should contain the evidence supporting the variant classification, including its predicted effect on the resultant protein and whether any variants identified are likely to fully or partially explain the patient's indication for testing. The report also should include any recommendations to the clinician for supplemental clinical testing, such as enzymatic/functional testing of the patient's cells and variant testing of family members, to further inform variant interpretation. The interpretation section should address all variants described in the results section but may contain additional information. It should be noted whether the variant has been reported previously in the literature or in disease or control databases. The references, if any, that contributed to the classification should be cited where discussed and listed at the end of the report. The additional information described in the interpretation section may include a summarized conclusion of the results of in silico analyses and evolutionary conservation analyses. However, individual computational predictions (e.g., scores, terms such as "damaging") should be avoided given the high likelihood of misinterpretation by health-care providers who may be unfamiliar with the limitations of predictive algorithms (see In Silico Predictive Programs, above). A discussion of decreased penetrance and variable expressivity of the disorder, if relevant, should be included in the final report. Examples of how to describe evidence for variant classification on clinical reports are found in the **Supplementary Appendix S1** online.

Methodology

The methods and types of variants detected by the assay and those refractory to detection should be provided in the report. Limitations of the assay used to detect the variants also should be reported. Methods should include those used to obtain nucleic acids (e.g., polymerase chain reaction, capture, whole-genome amplification), as well as those to analyze the nucleic acids (e.g., bidirectional Sanger sequencing, next-generation sequencing, chromosomal microarray, genotyping technologies), because this may provide the health-care provider with the necessary information to decide whether additional testing is required to follow up on the results. The methodology section should also give the official gene names approved by the Human Genome Organization Gene Nomenclature Committee, RefSeq accession numbers for transcripts, and genome build, including versions. For large panels, gene-level information may be posted and referenced by URL. The laboratory may choose to add a disclaimer that addresses general pitfalls in laboratory testing, such as sample quality and sample mix-up.

Access to patient advocacy groups, clinical trials, and research

Although specific clinical guidance for a patient is not recommended for laboratory reports, provision of general information for categories of results (e.g., all positives) is appropriate and helpful. A large number of patient advocacy groups and clinical trials are now available for support and treatment of many diseases. Laboratories may choose to add this information to the body of the report or attach the information so it is sent to the health-care provider along with the report.

Laboratories may make an effort to connect the health-care provider to research groups working on specific diseases when a variant's effect is classified as "uncertain," as long as Health Insurance Portability and Accountability Act patient privacy requirements are followed.

Variant reanalysis

As evidence on variants evolves, previous classifications may later require modification. For example, the availability of variant frequency data among large populations has led many uncertain significance variants to be reclassified as benign, and testing additional family members may result in the reclassification of variants.

As the content of sequencing tests expands and the number of variants identified grows, expanding to thousands and millions of variants from exome and genome sequencing, the ability for laboratories to update reports as variant knowledge changes will be untenable without appropriate mechanisms and resources to sustain those updates. To set appropriate expectations with health-care providers and patients, laboratories should provide clear policies on the reanalysis of data from genetic testing and whether additional charges for reanalysis may apply. Laboratories are encouraged to explore innovative approaches to give patients and providers more efficient access to updated information.[37,38]

For reports containing variants of uncertain significance in genes related to the primary indication, and in the absence of updates that may be proactively provided by the laboratory, it is recommended that laboratories suggest periodic inquiry by health-care providers to determine whether knowledge of any variants of uncertain significance, including variants reported as likely pathogenic, has changed. By contrast, laboratories are encouraged to consider proactive amendment of cases when a variant reported with a near-definitive classification (pathogenic or benign) must be reclassified. Regarding physician responsibility, see the ACMG guidelines on the duty to recontact.[39]

Confirmation of findings

Recommendations for the confirmation of reported variants is addressed elsewhere.[35,36] Except as noted, confirmation studies using an orthogonal method are recommended for all sequence variants that are considered to be pathogenic or likely pathogenic for a Mendelian disorder. These methods may include, but are not limited to, re-extraction of the sample and testing, testing of parents, restriction enzyme digestion, sequencing the area of interest a second time, or using an alternate genotyping technology.

SPECIAL CONSIDERATIONS

Evaluating and reporting variants in GUS based on the indication for testing

Genome and exome sequencing are identifying new genotype–phenotype connections. When the laboratory finds a variant in a gene without a validated association to the patient's phenotype, it is a GUS. This can occur when a gene has never been associated with any patient phenotype or when the gene has been associated with a different phenotype from that under consideration. Special care must be taken when applying the recommended guidelines to a GUS. In such situations, utilizing variant classification rules developed for recognized genotype–phenotype associations is not appropriate. For example, when looking across the exome or genome, a de novo observation is no longer strong evidence for pathogenicity given that all individuals are expected to have approximately one de novo variant in their exome or 100 in their genome. Likewise, thousands of variants across a genome could segregate with a significant logarithm of the odds (LOD) score. Furthermore, many deleterious variants that are clearly disruptive to a gene or its resultant protein (nonsense, frameshift, canonical ±1,2 splice site, exon-level deletion) may be detected; however, this is insufficient evidence for a causative role in any given disease presentation.

Variants found in a GUS may be considered as candidates and reported as "variants in a gene of uncertain significance." These variants, if reported, should always be classified as uncertain significance. Additional evidence would be required to support the gene's association to disease before any variant in the gene itself can be considered pathogenic for that disease.[5] For example, additional cases with matching rare phenotypes and deleterious variants in the same gene would enable the individual variants to be classified according to the recommendations presented here.

Evaluating variants in healthy individuals or as incidental findings

Caution must be exercised when using these guidelines to evaluate variants in healthy or asymptomatic individuals or to interpret incidental findings unrelated to the primary indication for testing. In these cases the likelihood of any identified variant being pathogenic may be far less than when performing disease-targeted testing. As such, the required evidence to call a variant pathogenic should be higher, and extra caution should be exercised. In addition, the predicted penetrance of pathogenic variants found in the absence of a phenotype or family history may be far less than predicted based on historical data from patients ascertained as having disease.

Mitochondrial variants

The interpretation of mitochondrial variants other than well-established pathogenic variants is complex and remains challenging; several special considerations are addressed here.

The nomenclature differs from standard nomenclature for nuclear genes, using gene name and m. numbering (e.g., m.8993T>C) and p. numbering, but not the standard c. numbering (see also Nomenclature). The current accepted reference sequence is the Revised Cambridge Reference Sequence of the Human Mitochondrial DNA: GenBank sequence NC_012920 gi:251831106.[40,41]

Heteroplasmy or homoplasmy should be reported, along with an estimate of heteroplasmy of the variant if the test has

been validated to determine heteroplasmy levels. Heteroplasmy percentages in different tissue types may vary from the sample tested; therefore, low heteroplasmic levels also must be interpreted in the context of the tissue tested, and they may be meaningful only in the affected tissue such as muscle. Over 275 mitochondrial DNA variants relating to disease have been recorded (http://mitomap.org/bin/view.pl/MITOMAP/WebHome).[42] MitoMap is considered the main source of information related to mitochondrial variants as well as haplotypes. Other resources, such as frequency information (http://www.mtdb.igp.uu.se/),[43] secondary structures, sequences, and alignment of mitochondrial transfer RNAs (http://mamit-trna.u-strasbg.fr/),[44] mitochondrial haplogroups (http://www.phylotree.org/)[45]and other information (http://www.mtdna-community.org/default.aspx),[46] may prove useful in interpreting mitochondrial variants.

Given the difficulty in assessing mitochondrial variants, a separate evidence checklist has not been included. However, any evidence needs to be applied with additional caution (for a review, see ref. [47]). The genes in the mitochondrial genome encode for transfer RNA as well as for protein; therefore, evaluating amino acid changes is relevant only for genes encoding proteins. Similarly, because many mitochondrial variants are missense variants, evidence criteria for truncating variants likely will not be helpful. Because truncating variants do not fit the known variant spectrum in most mitochondrial genes, their significance may be uncertain. Although mitochondrial variants are typically maternally inherited, they can be sporadic, yet de novo variants are difficult to assess because of heteroplasmy that may be below an assay's detection level or different between tissues. The level of heteroplasmy may contribute to the variable expression and reduced penetrance that occurs within families. Nevertheless, there remains a lack of correlation between the percentage of heteroplasmy and disease severity.[47] Muscle, liver, or urine may be additional specimen types useful for clinical evaluation. Undetected heteroplasmy may also affect outcomes of case, case–control, and familial concordance studies. In addition, functional studies are not readily available, although evaluating muscle morphology may be helpful (i.e., the presence of ragged red fibers). Frequency data and published studies demonstrating causality may often be the only assessable criteria on the checklist. An additional tool for mitochondrial diseases may be haplogroup analysis, but this may not represent a routine method that clinical laboratories have used, and the clinical correlation is not easy to interpret.

Consideration should be given to testing nuclear genes associated with mitochondrial disorders because variants in nuclear genes could be causative of oxidative disorders or modulating the mitochondrial variants.

Pharmacogenomics

Establishing the effects of variants in genes involved with drug metabolism is challenging, in part because a phenotype is only apparent upon exposure to a drug. Still, variants in genes related to drug efficacy and risk for adverse events have been described and are increasingly used in clinical care. Gene summaries and clinically relevant variants can be found in the Pharmacogenomics Knowledge Base (http://www.pharmgkb.org/).[48] Alleles and nomenclature for the cytochrome P450 gene family is available at http://www.cypalleles.ki.se/.[49] Although the interpretation of PGx variants is beyond the scope of this document, we include a discussion of the challenges and distinctions associated with the interpretation and reporting of PGx results.

The traditional nomenclature of PGx alleles uses star (*) alleles, which often represent haplotypes, or a combination of variants on the same allele. Traditional nucleotide numbering using outdated reference sequences is still being applied. Converting traditional nomenclature to standardized nomenclature using current reference sequences is an arduous task, but it is necessary for informatics applications with next-generation sequencing.

Many types of variants have been identified in PGx genes, such as truncating, missense, deletions, duplications (of functional as well as nonfunctional alleles), and gene conversions, resulting in functional, partially functional (decreased or reduced function), and nonfunctional (null) alleles. Interpreting sequence variants often requires determining haplotype from a combination of variants detected. Haplotypes are typically presumed based on population frequencies and known variant associations rather than testing directly for chromosomal phase (molecular haplotyping).

In addition, for many PGx genes (particularly variants in genes coding for enzymes), the overall phenotype is derived from a diplotype, which is the combination of variants or haplotypes on both alleles. Because PGx variants do not directly cause disease, using terms related to metabolism (rapid, intermediate, poor); efficacy (resistant, responsive, sensitive); or "risk," rather than pathogenic, may be more appropriate. Further nomenclature and interpretation guidelines are needed to establish consistency in this field.

Common complex disorders

Unlike Mendelian diseases, the identification of common, complex disease genes, such as those contributing to type 2 diabetes, coronary artery disease, and hypertension, has largely relied on population-based approaches (e.g., genome-wide association studies) rather than family-based studies.[50,51] Currently, numerous genome-wide association study reports have resulted in the cataloguing of over 1,200 risk alleles for common, complex diseases and traits. Most of these variants are in nongenic regions, however, and additional studies are required to determine whether any of the variants are directly causal through effects on regulatory elements, for example, or are in linkage disequilibrium with causal variants.[52]

Common, complex risk alleles typically confer low relative risk and are meager in their predictive power.[53] To date, the utility of common, complex risk allele testing for patient care[54] has been unclear, and models to combine multiple markers into a cumulative risk score often are flawed and are usually no better than traditional risk factors such as family

history, demographics, and nongenetic clinical phenotypes.[55,56] Moreover, in almost all of the common diseases the risk alleles can explain only up to 10% of the variance in the population, even when the disease has high heritability. Given the complexity of issues, this recommendation does not address the interpretation and reporting of complex trait alleles. We recognize, however, that some of these alleles are identified during the course of sequencing Mendelian genes, and therefore guidance on how to report such alleles when found incidentally is needed. The terms "pathogenic" and "likely pathogenic" are not appropriate in this context, even when the association is statistically valid. Until better guidance is developed, an interim solution is to report these variants as "risk alleles" or under a separate "other reportable" category in the diagnostic report. The evidence for the risk, as identified in the case–control/genome-wide association studies, can be expressed by modifying the terms, such as "established risk allele," "likely risk allele," or "uncertain risk allele," if desired.

Somatic variants

The description of somatic variants, primarily those observed in cancer cells, includes complexities not encountered with constitutional variants, because the allele ratios are highly variable and tumor heterogeneity can cause sampling differences. Interpretation helps select therapy and predicts treatment response or the prognosis of overall survival or tumor progression–free survival, further complicating variant classification. For the interpretation of negative results, understanding the limit of detection of the sequencing assay (at what allele frequency the variant can be detected by the assay) is important and requires specific knowledge of the tumor content of the sample. Variant classification categories are also different, with somatic variants compared with germ-line variants, with terms such as "responsive," "resistant," "driver," and "passenger" often used. Whether a variant is truly somatic is confirmed by sequence analysis of the patient's germ-line DNA. A different set of interpretation guidelines is needed for somatic variants, with tumor-specific databases used for reference, in addition to databases used for constitutional findings. To address this, a workgroup has recently been formed by the AMP.

HOW SHOULD HEALTH-CARE PROVIDERS USE THESE GUIDELINES AND RECOMMENDATIONS?

The primary purpose of clinical laboratory testing is to support medical decision making. In the clinic, genetic testing is generally used to identify or confirm the cause of disease and to help the health-care provider make individualized treatment decisions including the choice of medication. Given the complexity of genetic testing, results are best realized when the referring health-care provider and the clinical laboratory work collaboratively in the testing process.

When a health-care provider orders genetic testing, the patient's clinical information is integral to the laboratory's analysis. As health-care providers increasingly utilize genomic (exome or genome) sequencing, the need for detailed clinical information to aid in interpretation assumes increasing importance. For example, when a laboratory finds a rare or novel variant in a genomic sequencing sample, the director cannot assume it is relevant to a patient just because it is rare, novel, or de novo. The laboratory must evaluate the variant and the gene in the context of the patient's and family's history, physical examinations, and previous laboratory tests to distinguish between variants that cause the patient's disorder and those that are incidental (secondary) findings or benign. Indeed, accurate and complete clinical information is so essential for the interpretation of genome-level DNA sequence findings that the laboratory can reasonably refuse to proceed with the testing if such information is not provided with the test sample.

For tests that cover a broad range of phenotypes (large panels, exome and genome sequencing) the laboratory may find candidate causative variants. Further follow-up with the health-care provider and patient may uncover additional evidence to support a variant. These additional phenotypes may be subclinical, requiring additional clinical evaluation to detect (e.g., temporal bone abnormalities detected by computed tomography in a hearing-impaired patient with an uncertain variant in *SLC26A4*, the gene associated with Pendred syndrome). In addition, testing other family members to establish when a variant is de novo, when a variant cosegregates with disease in the family, and when a variant is in *trans* with a pathogenic variant in the same recessive disease-causing gene is valuable. Filtering out or discounting the vast majority of variants for dominant diseases when they can be observed in healthy relatives is possible, making the interpretation much more efficient and conclusive. To this end, it is strongly recommended that every effort be made to include parental samples along with that of the proband, so-called "trio" testing (mother, father, affected child), in the setting of exome and genome sequencing, particularly for suspected recessive or de novo causes. Obviously this will be easier to achieve for pediatric patients than for affected adults. In the absence of one or both parents, the inclusion of affected and unaffected siblings can be of value.

Many genetic variants can result in a range of phenotypic expression (variable expressivity), and the chance of disease developing may not be 100% (reduced penetrance), further underscoring the importance of providing comprehensive clinical data to the clinical laboratory to aid in variant interpretation. Ideally, it is recommended that clinical data be deposited into, and shared via, centralized repositories as allowable by Health Insurance Portability and Accountability Act and institutional review board regulations. Importantly, referring health-care providers can further assist clinical laboratories by recruiting DNA from family members in scenarios where their participation will be required to interpret results, (e.g., when evaluating cosegregation with disease using affected family members, genotyping parents to assess for de novo occurrence and determining the phase of variants in recessive disorders using first-degree relatives).

A key issue for health-care providers is how to use the evidence provided by genetic testing in medical management decisions. Variant analysis is, at present, imperfect, and the variant category reported does not imply 100% certainty. In general, a variant classified as pathogenic using the proposed classification scheme has met criteria informed by empirical data such that a health-care provider can use the molecular testing information in clinical decision making. Efforts should be made to avoid using this as the sole evidence of Mendelian disease; it should be used in conjunction with other clinical information when possible. Typically, a variant classified as likely pathogenic has sufficient evidence that a health-care provider can use the molecular testing information in clinical decision making when combined with other evidence of the disease in question. For example, in the prenatal setting an ultrasound may show a key confirmatory finding; in postnatal cases, other data such as enzyme assays, physical findings, or imaging studies may conclusively support decision making. However, it is recommended that all possible follow-up testing, as described above, be pursued to generate additional evidence related to a likely pathogenic variant because this may permit the variant to be reclassified as pathogenic. A variant of uncertain significance should not be used in clinical decision making. Efforts to resolve the classification of the variant as pathogenic or benign should be undertaken. While this effort to reclassify the variant is underway, additional monitoring of the patient for the disorder in question may be prudent. A variant considered likely benign has sufficient evidence that a health-care provider can conclude that it is not the cause of the patient's disorder when combined with other information, for example, if the variant does not segregate in an affected family member and complex inheritance patterns are unlikely. A variant considered benign has sufficient evidence that a health-care provider can conclude that it is not the cause of the patient's disorder.

How the genetic testing evidence is used is also dependent on the clinical context and indication for testing. In a prenatal diagnostic case where a family is considering irrevocable decisions such as fetal treatment or pregnancy termination, the weight of evidence from the report and other sources such as fetal ultrasound needs to be considered before action is taken. When a genetic test result is the *only* evidence in a prenatal setting, variants considered likely pathogenic must be explained carefully to families. It is therefore critical for referring health-care providers to communicate with the clinical laboratory to gain an understanding of how variants are classified to assist in patient counseling and management.

SUPPLEMENTARY MATERIAL

Supplementary material is linked to the online version of the paper at http://www.nature.com/gim

ACKNOWLEDGMENTS

The workgroup thanks our many colleagues in the genetics community who helped to develop this "consensus" guideline through their responses to surveys, participation at workshops, insightful discussions, and constructive comments. H.L.R., S.B., S.D., M.H., and E.L. were supported in part by National Institutes of Health HG006834. H.L.R. was supported in part by National Institutes of Health grants HG006500 and HD077671. These Standards and Guidelines were approved by the ACMG Board of Directors on 15 December 2014 and the AMP Board of Directors on 9 January 2015.

DISCLOSURE

All workgroup members are clinical service providers. The following workgroup members have a commercial conflict of interest: S.B. (GeneDx, BioReference (stock), advisory boards for RainDance, Ingenuity); M.H. (advisor for Oxford Genetic Technologies, Tessarae, Ingenuity/Qiagen); E.L. (advisory board for Complete Genomics); and H.L.R. (scientific advisory boards for Ingenuity/Qiagen, Complete Genomics, Knome, Focused Genomics). The other authors declare no conflict of interest.

REFERENCES

1. Richards CS, Bale S, Bellissimo DB, et al.; Molecular Subcommittee of the ACMG Laboratory Quality Assurance Committee. ACMG recommendations for standards for interpretation and reporting of sequence variations: Revisions 2007. *Genet Med* 2008;10:294–300.
2. Plon SE, Eccles DM, Easton D, et al.; IARC Unclassified Genetic Variants Working Group. Sequence variant classification and reporting: recommendations for improving the interpretation of cancer susceptibility genetic test results. *Hum Mutat* 2008;29:1282–1291.
3. Sosnay PR, Siklosi KR, Van Goor F, et al. Defining the disease liability of variants in the cystic fibrosis transmembrane conductance regulator gene. *Nat Genet* 2013;45:1160–1167.
4. Thompson BA, Spurdle AB, Plazzer JP, et al.; InSiGHT. Application of a 5-tiered scheme for standardized classification of 2,360 unique mismatch repair gene variants in the InSiGHT locus-specific database. *Nat Genet* 2014;46:107–115.
5. MacArthur DG, Manolio TA, Dimmock DP, et al. Guidelines for investigating causality of sequence variants in human disease. *Nature* 2014;508:469–476.
6. Kearney HM, Thorland EC, Brown KK, Quintero-Rivera F, South ST; Working Group of the American College of Medical Genetics Laboratory Quality Assurance Committee. American College of Medical Genetics standards and guidelines for interpretation and reporting of postnatal constitutional copy number variants. *Genet Med* 2011;13:680–685.
7. Nomenclature for the description of sequence variants: Human Genome Variation Society. https://www.hgvs.org/mutnomen. Accessed 4 November 2014.
8. Wildeman M, van Ophuizen E, den Dunnen JT, Taschner PE. Improving sequence variant descriptions in mutation databases and literature using the Mutalyzer sequence variation nomenclature checker. *Hum Mutat* 2008;29:6–13.
9. RefSeq: NCBI Reference Sequence Database. https://www.ncbi.nlm.nih.gov/refseq. Accessed 4 November 2014.
10. Locus Reference Genomic. https://www.lrg-sequence.org. Accessed 4 November 2014.
11. Concensus CDS Database NCBI. https://www.ncbi.nlm.nih.gov/CCDS/CcdsBrowse.cgi. Accessed 4 November 2014.
12. Taschner PE, den Dunnen JT. Describing structural changes by extending HGVS sequence variation nomenclature. *Hum Mutat* 2011;32:507–511.
13. Kircher M, Witten DM, Jain P, O'Roak BJ, Cooper GM, Shendure J. A general framework for estimating the relative pathogenicity of human genetic variants. *Nat Genet* 2014;46:310–315.
14. Hicks S, Wheeler DA, Plon SE, Kimmel M. Prediction of missense mutation functionality depends on both the algorithm and sequence alignment employed. *Hum Mutat* 2011;32:661–668.
15. Tavtigian SV, Greenblatt MS, Lesueur F, Byrnes GB; IARC Unclassified Genetic Variants Working Group. In silico analysis of missense substitutions using sequence-alignment based methods. *Hum Mutat* 2008;29:1327–1336.
16. Thusberg J, Olatubosun A, Vihinen M. Performance of mutation pathogenicity prediction methods on missense variants. *Hum Mutat* 2011;32:358–368.

17. Thompson BA, Greenblatt MS, Vallee MP, et al. Calibration of multiple in silico tools for predicting pathogenicity of mismatch repair gene missense substitutions. *Hum Mutat* 2013;34:255–265.
18. Choi Y, Sims GE, Murphy S, Miller JR, Chan AP. Predicting the functional effect of amino acid substitutions and indels. *PLoS One* 2012;7:e46688.
19. Adzhubei IA, Schmidt S, Peshkin L, et al. A method and server for predicting damaging missense mutations. *Nat Methods* 2010;7:248–249.
20. Kumar P, Henikoff S, Ng PC. Predicting the effects of coding non-synonymous variants on protein function using the SIFT algorithm. *Nat Protoc* 2009;4:1073–1081.
21. Schwarz JM, Rödelsperger C, Schuelke M, Seelow D. MutationTaster evaluates disease-causing potential of sequence alterations. *Nat Methods* 2010;7:575–576.
22. Jian X, Boerwinkle E, Liu X. In silico tools for splicing defect prediction: a survey from the viewpoint of end users. *Genet Med* 2014;16:497–503.
23. Houdayer C, Caux-Moncoutier V, Krieger S, et al. Guidelines for splicing analysis in molecular diagnosis derived from a set of 327 combined in silico/in vitro studies on BRCA1 and BRCA2 variants. *Hum Mutat* 2012;33:1228–1238.
24. Vreeswijk MP, Kraan JN, van der Klift HM, et al. Intronic variants in BRCA1 and BRCA2 that affect RNA splicing can be reliably selected by splice-site prediction programs. *Hum Mutat* 2009;30:107–114.
25. Henderson LB, Applegate CD, Wohler E, Sheridan MB, Hoover-Fong J, Batista DA. The impact of chromosomal microarray on clinical management: a retrospective analysis. *Genet Med* 2014;16:657–664.
26. Popp MW, Maquat LE. Organizing principles of mammalian nonsense-mediated mRNA decay. *Annu Rev Genet* 2013;47:139–165.
27. Calculator for confidence intervals of odds ratio in an unmatched case control study. For example groups of cases and controls studied to assess a treatment or exposure to a suspected causal factor. http://www.hutchon.net/confidor.htm. Accessed 4 November 2014.
28. Odds Ratio Confidence Interval Calculator. http://www.easycalculation.com/statistics/odds-ratio. Accessed 4 November 2014.
29. Bayrak-Toydemir P, McDonald J, Mao R, et al. Likelihood ratios to assess genetic evidence for clinical significance of uncertain variants: hereditary hemorrhagic telangiectasia as a model. *Exp Mol Pathol* 2008;85:45–49.
30. Thompson D, Easton DF, Goldgar DE. A full-likelihood method for the evaluation of causality of sequence variants from family data. *Am J Hum Genet* 2003;73:652–655.
31. Goldgar DE, Easton DF, Deffenbaugh AM, Monteiro AN, Tavtigian SV, Couch FJ; Breast Cancer Information Core (BIC) Steering Committee. Integrated evaluation of DNA sequence variants of unknown clinical significance: application to BRCA1 and BRCA2. *Am J Hum Genet* 2004;75:535–544.
32. Scheuner MT, Hilborne L, Brown J, Lubin IM; members of the RAND Molecular Genetic Test Report Advisory Board. A report template for molecular genetic tests designed to improve communication between the clinician and laboratory. *Genet Test Mol Biomarkers* 2012;16:761–769.
33. Lubin IM, Caggana M, Constantin C, et al. Ordering molecular genetic tests and reporting results: practices in laboratory and clinical settings. *J Mol Diagn* 2008;10:459–468.
34. Lubin IM, McGovern MM, Gibson Z, et al. Clinician perspectives about molecular genetic testing for heritable conditions and development of a clinician-friendly laboratory report. *J Mol Diagn* 2009;11:162–171.
35. Rehm HL, Bale SJ, Bayrak-Toydemir P, et al.; Working Group of the American College of Medical Genetics and Genomics Laboratory Quality Assurance Commitee. ACMG clinical laboratory standards for next-generation sequencing. *Genet Med* 2013;15:733–747.
36. Aziz N, Zhao Q, Bry L, et al. College of American Pathologists' Laboratory Standards for Next-Generation Sequencing Clinical Tests. *Arch Pathol Lab Med* 2014. doi:10.5858/arpa.2014-0250-CP.
37. Aronson SJ, Clark EH, Varugheese M, Baxter S, Babb LJ, Rehm HL. Communicating new knowledge on previously reported genetic variants. *Genet Med* 2012;14:713–719.
38. Bean LJ, Tinker SW, da Silva C, Hegde MR. Free the data: one laboratory's approach to knowledge-based genomic variant classification and preparation for EMR integration of genomic data. *Hum Mutat* 2013;34:1183–1188.
39. Hirschhorn K, Fleisher LD, Godmilow L, et al. Duty to re-contact. *Genet Med* 1999;1:171–172.
40. Revised Cambridge Reference Sequence (rCRS) of the Human Mitochondrial DNA: GenBank sequence NC_012920 gi:251831106. http://www.mitomap.org/MITOMAP/HumanMitoSeq.
41. Behar DM, van Oven M, Rosset S, et al. A "Copernican" reassessment of the human mitochondrial DNA tree from its root. *Am J Hum Genet* 2012;90:675–684.
42. MITOMAP: A human mitochondrial genome database. http://mitomap.org/bin/view.pl/MITOMAP/WebHome. Accessed 4 November 2014.
43. mtDB-Human Mitochondrial Genome Database. http://www.mtdb.igp.uu.se. Accessed 4 November 2014.
44. Compilation of mammalian mitochondrial tRNA genes. http://mamit-trna.u-strasbg.fr/Summary.asp. Accessed 4 November 2014.
45. van Oven M, Kayser M. Updated comprehensive phylogenetic tree of global human mitochondrial DNA variation. *Hum Mutat* 2009;30:E386–E394.
46. mtDNA Community. http://www.mtdnacommunity.org/default.aspx/. Accessed 4 November 2014.
47. Wong LJ. Diagnostic challenges of mitochondrial DNA disorders. *Mitochondrion* 2007;7:45–52.
48. PharmGKB. http://www.pharmgkb.org. Accessed 4 November 2014.
49. The Human Cytochrome P450 (CYP) Allele Nomenclature Database. http://www.cypalleles.ki.se/. Accessed 4 November 2014.
50. Manolio TA. Genomewide association studies and assessment of the risk of disease. *N Engl J Med* 2010;363:166–176.
51. Hardy J, Singleton A. Genomewide association studies and human disease. *N Engl J Med* 2009;360:1759–1768.
52. Kruglyak L. Prospects for whole-genome linkage disequilibrium mapping of common disease genes. *Nat Genet* 1999;22:139–144.
53. McClellan J, King MC. Genetic heterogeneity in human disease. *Cell* 2010;141:210–217.
54. Topol EJ, Damani SB. The KIF6 collapse. *J Am Coll Cardiol* 2010;56:1564–1566.
55. Meigs JB, Shrader P, Sullivan LM, et al. Genotype score in addition to common risk factors for prediction of type 2 diabetes. *N Engl J Med* 2008;359:2208–2219.
56. Paynter NP, Chasman DI, Paré G, et al. Association between a literature-based genetic risk score and cardiovascular events in women. *JAMA* 2010;303:631–637.
57. Kerem E, Corey M, Kerem BS, et al. The relation between genotype and phenotype in cystic fibrosis–analysis of the most common mutation (delta F508). *N Engl J Med*. 1990 Nov 29;323(22):1517–22.
58. Kobayashi K, Knowles MR, Boucher RC, et al. Benign missense variations in the cystic fibrosis gene. *Am J Hum Genet*. 1990 Oct;47(4):611–615.
59. Zelante L, Gasparini P, Estivill X, et al. Connexin26 mutations associated with the most common form of non-syndromic neurosensory autosomal recessive deafness (DFNB1) in Mediterraneans. *Hum Mol Genet*. 1997 Sep;6(9):1605–9.
